# Autologous and Allogeneic Cytotherapies for Large Knee (Osteo)Chondral Defects: Manufacturing Process Benchmarking and Parallel Functional Qualification

**DOI:** 10.3390/pharmaceutics15092333

**Published:** 2023-09-16

**Authors:** Virginie Philippe, Annick Jeannerat, Cédric Peneveyre, Sandra Jaccoud, Corinne Scaletta, Nathalie Hirt-Burri, Philippe Abdel-Sayed, Wassim Raffoul, Salim Darwiche, Lee Ann Applegate, Robin Martin, Alexis Laurent

**Affiliations:** 1 Orthopedics and Traumatology Service, Lausanne University Hospital, University of Lausanne, CH-1011 Lausanne, Switzerland; robin.martin@chuv.ch; 2 Regenerative Therapy Unit, Plastic, Reconstructive and Hand Surgery Service, Lausanne University Hospital, University of Lausanne, CH-1066 Epalinges, Switzerland; sandra.jaccoud@chuv.ch (S.J.); corinne.scaletta@chuv.ch (C.S.); nathalie.burri@chuv.ch (N.H.-B.); philippe.abdel-sayed@chuv.ch (P.A.-S.); wassim.raffoul@chuv.ch (W.R.); lee.laurent-applegate@chuv.ch (L.A.A.); 3 Preclinical Research Department, LAM Biotechnologies SA, CH-1066 Epalinges, Switzerland; annick.jeannerat@lambiotechnologies.com (A.J.); cedric.peneveyre@lambiotechnologies.com (C.P.); 4 Laboratory of Biomechanical Orthopedics, Federal Polytechnic School of Lausanne, CH-1015 Lausanne, Switzerland; 5 STI School of Engineering, Federal Polytechnic School of Lausanne, CH-1015 Lausanne, Switzerland; 6 Musculoskeletal Research Unit, Vetsuisse Faculty, University of Zurich, CH-8057 Zurich, Switzerland; sdarwiche@vetclinics.uzh.ch; 7 Center for Applied Biotechnology and Molecular Medicine, University of Zurich, CH-8057 Zurich, Switzerland; 8 Oxford OSCAR Suzhou Center, Oxford University, Suzhou 215123, China

**Keywords:** allogeneic cytotherapies, autologous chondrocyte implantation, cartilage defect, chondrogenesis, cell therapy, FE002 primary chondroprogenitors, manufacturing process, standardized transplant product, tissue engineering, translational research

## Abstract

Cytotherapies are often necessary for the management of symptomatic large knee (osteo)-chondral defects. While autologous chondrocyte implantation (ACI) has been clinically used for 30 years, allogeneic cells (clinical-grade FE002 primary chondroprogenitors) have been investigated in translational settings (Swiss progenitor cell transplantation program). The aim of this study was to comparatively assess autologous and allogeneic approaches (quality, safety, functional attributes) to cell-based knee chondrotherapies developed for clinical use. Protocol benchmarking from a manufacturing process and control viewpoint enabled us to highlight the respective advantages and risks. Safety data (telomerase and soft agarose colony formation assays, high passage cell senescence) and risk analyses were reported for the allogeneic FE002 cellular active substance in preparation for an autologous to allogeneic clinical protocol transposition. Validation results on autologous bioengineered grafts (autologous chondrocyte-bearing Chondro-Gide scaffolds) confirmed significant chondrogenic induction (*COL2* and *ACAN* upregulation, extracellular matrix synthesis) after 2 weeks of co-culture. Allogeneic grafts (bearing FE002 primary chondroprogenitors) displayed comparable endpoint quality and functionality attributes. Parameters of translational relevance (transport medium, finished product suturability) were validated for the allogeneic protocol. Notably, the process-based benchmarking of both approaches highlighted the key advantages of allogeneic FE002 cell-bearing grafts (reduced cellular variability, enhanced process standardization, rationalized logistical and clinical pathways). Overall, this study built on our robust knowledge and local experience with ACI (long-term safety and efficacy), setting an appropriate standard for further clinical investigations into allogeneic progenitor cell-based orthopedic protocols.

## 1. Introduction

Patients presenting symptomatic large knee cartilage lesions often report pain, swelling, joint locking, stiffness, and clicking [1,2,3,4]. The resulting functional impairments often negatively impact daily life activities, and untreated cartilage lesions predispose for osteoarthritis (OA) [4,5,6]. Therefore, early treatment for the restoration of cartilage structure and function could lead to measurable benefits for patient quality of life and tangibly limit the progression of OA [4,6]. However, articular cartilage is characterized by poor self-healing potential due to low vascularity and a limited supply of adjacent cells able to migrate to the lesion and mediate a healing response [2,3,6]. Therefore, various therapeutic approaches have been developed for the treatment of moderate knee chondropathies, such as microfracture (MFx) or osteochondral autografts and allografts, yet their use and efficacy are limited by the lesion type, size, and grade [7,8,9,10,11,12,13,14]. Importantly, the onset of severe OA often leads to the need to replace the arthritic surface with a synthetic prosthesis. While this approach is the current standard in older sedentary patients, it is less desirable for active and young patients [4,6].

From a specific cytotherapeutic viewpoint, Brittberg et al. have reported autologous chondrocyte implantation (ACI) clinical protocols for articular cartilage defect treatment since 1994 [3,7,8,15,16,17,18]. Applications of cultured autologous chondrocytes aim to promote the restauration of hyaline cartilage, providing the structural and biomechanical properties required to sustain normal joint load-bearing and function [1,2,7,11]. Despite documented inhomogeneity in cell therapy manufacturing processes and surgical approaches, the accumulated evidence points toward a beneficial effect of ACI over MFx in the medium and long term [5,9,16,18,19,20,21,22,23,24,25,26]. Since the initial reports on ACI, thousands of patients have been successfully treated, and the technique has evolved to further improve clinical outcomes [8,14,27,28,29,30,31]. Specifically, third-generation ACI involves the use of a synthetic scaffold/matrix for autologous graft bioengineering before surgical implantation [11,12,21,24,26,31,32,33,34,35,36,37]. This approach involves less invasive surgical procedures, and modern synthetic scaffolds are less fragile than the periosteal flaps used in previous ACI generations [2,8,38,39,40]. In addition to improved therapeutic cell localization, the 3D scaffold-based co-culture step induces internal re-differentiation and the expression of specific chondrogenic genes such as *COL2* and *ACAN* [31,41,42,43,44,45,46,47].

Whilst ACI has been clinically investigated for three decades and yielded significant beneficial results, the technique is scarcely implanted, mainly due to limited good manufacturing practice (GMP)-compliant production capabilities [4,48]. Therefore, building on our in-house translational experience regarding second-generation ACI (i.e., NCT04296487 clinical trial), a local multi-centric clinical study was approved for third-generation ACI (i.e., NCT05651997 clinical trial) [8,11,12,26,35,48]. Specifically, the studied autologous chondral graft is indicated for large ICRS grade III or IV localized and symptomatic knee cartilage lesions [4,48]. Parallelly to this autologous approach, multifaceted translational work was carried out under the Swiss progenitor cell transplantation program for the eventual cytotherapeutic use of allogeneic primary chondroprogenitors (i.e., FE002 clinical-grade cells) in human orthopedics [49,50,51,52,53,54]. Given that both tissue engineering approaches share technical, clinical, and regulatory similarities, both options are currently being locally investigated [48,50].

Generally, various therapeutic cell sources (e.g., mesenchymal stem cells, infant polydactyly chondrocytes, genetically engineered cells) were considered up to the clinical investigation and post-market follow-up (FU) stages of cartilage regenerative medicine [10,11,33,55,56,57,58,59,60,61,62,63,64]. Importantly, FE002 primary chondroprogenitors have been established as a standardized clinical-grade cell source and preclinically qualified for tissue engineering purposes [49,50,51,52,53]. Ten years of multicentric translational research and their good safety profile have validated the robustness and versatility of FE002 progenitor cells for industrial cytotherapeutic formulation in cartilage tissue engineering [50,51,52,53]. Specifically, clinical-grade FE002 cell source establishment and upscaled cell manufacturing optimization have validated their adequation with standard industrial biotechnology and biobanking workflows [50]. Importantly, the technical aspects of manufacturing and clinical safety risk analyses regarding FE002 primary chondroprogenitors were based on the international clinical uses of alternative FE002 progenitor cell sources [65]. Overall, several advantages were outlined regarding the considered cytotherapeutic use of FE002 primary chondroprogenitors over autologous chondrocytes, such as reduced operative burdens and optimized serial bioengineered graft manufacturing possibilities [50].

Therefore, the objective of this study was to comparatively assess the autologous and allogeneic approaches to large knee (osteo)-chondral defect cytotherapeutic management that have been locally developed for clinical application. The translational significance of this work lies in the use of a regulatorily validated autologous somatic cell therapy protocol as the baseline for benchmarking with a novel allogeneic approach valorizing a clinical-grade allogeneic FE002 progenitor cell source. Our specific areas of experimental focus included the comparative translational qualification and validation of combined cytotherapeutic product quality, safety, and functional attributes. The benchmarking of both protocols for chondral graft preparation from a manufacturing process and control viewpoint enabled us to discuss the respective opportunities, advantages, and risks of each approach. Overall, this study set forth the robust research and local clinical experience reported with respect to ACI for large knee (osteo)-chondral defects. By extension, this work also enabled the authors to establish an appropriate continuum for further local clinical investigations into cell-based orthopedic protocols, with a specific focus on autologous to allogeneic approach transposition.

## 2. Materials and Methods

### 2.1. Reagents and Consumables Used for the Study

The reagents and consumables were as follows: purified water (Bichsel, Unterseen, Switzerland); DMEM cell culture medium, L-glutamine, TrypLE, Opti-MEM, penicillin-streptomycin, dexamethasone, TRIzol, BCA assay kits, NuPAGE Bis-Tris 4–12% protein gels, MOPS buffer, loading buffer, DTT, gel migration buffer antioxidant, page ruler protein ladder, MTT, β-mercaptoethanol, PMSF, microAmp fast 96-well reaction plates, optical adhesive covers, and 96-well PCR plates (Thermo Fisher Scientific, Waltham, MA, USA); C-Chip Neubauer hemocytometers (NanoEntek, Seoul, Republic of Korea); KAPA SYBR Fast (Roche, Basel, Switzerland); PrimeScript RT reagent kits (Takara Bio, San Jose, CA, USA); X-gal powder (Chemie Brunschwig, Basel, Switzerland); HAM’s F12 nutrient mix and papain (Sigma Aldrich, Buchs, Switzerland); Millipore Stericup, Trypan Blue, FBS, VitCp, sodium acetate, EDTA, low-melting point agarose, and cysteine HCl (Merck, Darmstadt, Germany); HPL (Sexton Biotechnologies, Indianapolis, IN, USA); human insulin (Novo Nordisk, Bagsværd, Danemark); ascorbic acid (Streuli Pharma, Uznach, Switzerland); ITS 100× (PAN-Biotech, Aidenbach, Germany); cell culture vessels, assay tubes, and plastic assay surfaces (Greiner BioOne, Frickenhausen, Germany; Corning, Corning, NY, USA; TPP, Trasadingen, Switzerland); TGF-β1 and TGF-β3 (PeproTech, London, UK); Chondro-Gide membranes (Geistlich Pharma, Wolhusen, Switzerland); Hyalograft membranes (Anika Therapeutics, Bedford, MA, USA); Blyscan-sulfated glycosaminoglycan assay kits (BioColor, Carrickfergus, UK); telomerase activity quantification qPCR assay kits (ScienCell, Carlsbad, CA, USA); Monosyn 6/0 suture kits (B. Braun, Melsungen, Germany).

### 2.2. Instruments and Equipment Used for the Study

Component weighing was performed using a laboratory scale (Ohaus, Parsippany, NJ, USA). Sample centrifugation was performed using a Rotina 420R centrifuge (Hettich, Tuttlingen, Germany) or on a Sorvall Legend Micro 21R microcentrifuge (Thermo Fisher Scientific, Waltham, MA, USA). Colorimetric and luminescence measurements were taken using a Varioskan LUX multimode plate reader (Thermo Fisher Scientific, Waltham, MA, USA). Telomerase activity and chondrogenic gene expression quantification assays were run on a StepOne Real-time PCR System instrument (Thermo Fisher Scientific, Waltham, MA, USA). Spectrophotometric analyses were performed using a NanoDrop instrument (Thermo Fisher Scientific, Waltham, MA, USA). Immunohistochemistry imaging was performed using an inverted IX81 fluorescence microscope (Olympus, Tokyo, Japan).

### 2.3. Ethical Compliance of the Study and Regulatory Approval of Investigative Cytotherapeutic Protocols

This study was performed using autologous biological materials (patient primary articular chondrocytes) gathered in the context of an authorized clinical trial (www.ClinicalTrials.gov, accessed on 4 August 2023, identifier NCT04296487, “Introduction of ACI for Cartilage Repair”, Lausanne, Switzerland) and/or included in the Biobank of the Department of Musculoskeletal Medicine in the Lausanne University Hospital [48]. Appropriate data security protocols were followed during the study. The described biological materials were used within the registration process of a second clinical trial (NCT05651997, “Study Comparing Two Methods for the Treatment of Large Chondral and Osteochondral Defects of the Knee”, Lausanne and Fribourg, Switzerland). The second clinical trial was approved by the local cantonal ethics committee (Vaud Cantonal Ethics Committee, CER-VD authorization N°2020-01707). The corresponding clinical trial was registered following federal authorization by Swissmedic (authorization N°2021TpP2004).

The allogeneic FE002 primary chondroprogenitor cell source used in the study (the clinical-grade FE002 progenitor cell source) was established from a registered organ donation, as approved by the Vaud Cantonal Ethics Committee (Ethics Committee Protocol N°62/07). The FE002 organ donation was registered under a federal cell transplantation program (the Swiss progenitor cell transplantation program) [50]. Full material traceability was ensured during the study.

### 2.4. Autologous Primary Chondrocyte Sourcing and Chondrogenic Cellular Active Substance Lot Manufacturing

The clinical-grade autologous primary chondrocytes (human articular chondrocytes [HAC] from orthopedic patients) used for the study consisted of banked human diploid cells. Cell type establishment was performed following cartilage biopsy procurement by the Orthopedics and Traumatology Service of the Lausanne University Hospital (Lausanne, Switzerland). The cryopreserved HACs were obtained from the biobank of the Department of Musculoskeletal Medicine in the Lausanne University Hospital. Patient-specific primary cell type establishment was performed in-house as described previously [48]. The HACs were manufactured using serial in vitro cellular expansions and were used in the present study at passage levels 3–4.

Briefly, the harvested healthy cartilage biopsies were rinsed, manually fragmented, and enzymatically treated (using pronase/collagenase) for HAC isolation. Following sample filtration on a cell sieve, the cell suspensions were expanded in vitro using human platelet lysate (HPL)-enriched culture medium (DMEM–HAM’s F12 base). After 1–2 cell passage procedures, the expanded HACs were formulated for cryopreservation and stored until further use [48]. The described processes were approved by the relevant health authorities in the framework of a clinical trial (NCT04296487).

### 2.5. Allogeneic FE002 Primary Chondroprogenitor Sourcing and Chondrogenic Cellular Active Substance Lot Manufacturing

The FE002 primary chondroprogenitor cell source used for the study consisted of banked primary human diploid cells from a clinical-grade source, as previously described [50]. The considered FE002 primary progenitor cells were procured and produced under the Swiss progenitor cell transplantation program and were made available as cryopreserved stocks. The FE002 primary chondroprogenitors were manufactured using a serial in vitro cellular expansion workflow and were used in the study at in vitro passages 6–7. Briefly, the cryopreserved cell vials were used as cellular seeding materials for in vitro monolayer expansions. Following thawing, the cells were cultured in fetal bovine serum (FBS)-enriched or HPL-enriched cell culture medium (DMEM base). Following the in vitro monolayer expansion phase, the expanded cells were formulated for cryopreservation and stored until further use [50].

### 2.6. FE002 Primary Chondroprogenitor Cellular Active Substance Characterization Assays

Extensive characterization and qualification work was already reported for the considered clinical-grade FE002 primary chondroprogenitors [49,50,51,52,53]. Specifically, the cellular active substance quality-related attributes and technical specifications for industrial scale cell bank manufacture had already been published [50]. Additionally, several in vivo studies have been performed on bioengineered product prototypes bearing viable allogeneic FE002 primary chondroprogenitors, confirming their safety and functionality in the retained setups [49,50,51,52,53]. Therefore, primarily based on a gap analysis of the safety characterization of FE002 primary chondroprogenitors, several in vitro assays were conducted for this study to further confirm their applicability in a translational setting.

#### 2.6.1. Allogeneic FE002 Cellular Active Substance Characterization: Cell Expansion Medium Selection

The previously reported work on FE002 primary chondroprogenitors was performed using FBS-supplemented cell proliferation medium [50]. In order to investigate whether alternative cell proliferation media could be used while maintaining cellular quality attributes, comparative proliferation assays were performed. Firstly, three different cell proliferation media (10% FBS in DMEM; 5% HPL in DMEM; Brittberg medium) and two different cell culture surfaces were used to assess the robustness of FE002 primary chondroprogenitors in culture. The Brittberg medium was composed of DMEM–HAM’s F12 (1:1) with 2 mM L-glutamine and 25 μg/mL ascorbic acid. Culture vessel incubation was performed in humidified incubators under 5% CO_2_ at 37 °C, and the cell proliferation medium was exchanged twice weekly. Operator assessments were performed regularly and recorded. Secondly, quantitative proliferation assays were performed using various HPL concentrations with a 10% FBS control, and the corresponding growth curves were recorded.

#### 2.6.2. Allogeneic FE002 Cellular Active Substance Characterization: Multiplex Proteomic Analyses

Soluble protein quantification was performed for the cellular materials of interest using specific multiplex analyses (Eve Technologies, Calgary, AB, Canada). The analyses (Discovery Assays) comprised the human angiogenesis array and growth factor 17-plex array, the human cytokine/chemokine 65-plex panel, the human-soluble cytokine receptor 14-plex array, the human MMP (matrix metalloproteinases) and TIMP (tissue inhibitors of metalloproteinases) panel for cell cultures, the cytokine TGF-β (transforming growth factor-β) 3-plex array, and the HFNFS-04 array. Briefly, the samples were prepared using bulk FE002 primary chondroprogenitor lysate with 10^7^ cell equivalents/mL. The samples were centrifuged at 13,000 rpm at 4 °C for 5 min. The resulting supernatants were frozen and shipped on dry ice for proteomic analyses. Simultaneously, the total protein contents of the samples were determined using colorimetric BCA assay kits and following the manufacturer’s instructions.

#### 2.6.3. Allogeneic FE002 Cellular Active Substance Qualification: β-Galactosidase Staining for In Vitro Senescence Assessment

An in vitro β-galactosidase assay (i.e., cellular senescence marker) was performed to confirm that FE002 primary chondroprogenitors did not yet reach senescence in culture at a passage level superior to that used in the allogeneic chondral grafts (i.e., passages 7–8). Briefly, FE002 primary chondroprogenitors were seeded in T25 cell culture flasks at 1.5 × 10^3^ cells/cm^2^ and expanded until they reached 70% confluency. Culture vessel incubation was performed in humidified incubators under 5% CO_2_ at 37 °C, and the cell proliferation medium was exchanged twice weekly. The cells were then fixed for 5 min in 10 mL of fixation solution containing 1.85% formaldehyde with 0.2% glutaraldehyde. The cells were then rinsed twice using PBS. The cells were stained overnight at 37 °C with a SA-β-gal staining solution consisting of 0.1% X-gal, 5 mM potassium ferrocyanide, 5 mM potassium ferricyanide, 150 mM NaCl, and 2 mM MgCl_2_ in a 40 mM citric acid/sodium phosphate solution at pH 6.0. The cells were washed twice with PBS and once with DMSO to remove the staining solution. The presence of β-galactosidase-positive (i.e., blue staining) cells was observed microscopically using 40× and 100× magnification. Random field acquisition was performed using the same microscopy setup, and the obtained images were used for β-galactosidase-positive cell operator enumeration.

#### 2.6.4. Allogeneic FE002 Cellular Active Substance Qualification: Telomerase Activity Quantification for In Vitro Tumorigenicity Assessment

An in vitro telomerase assay was performed using a qPCR telomerase activity quantification kit to confirm the non-tumorigenic potential of FE002 primary chondroprogenitors. Telomerase activity quantification was performed using frozen cellular dry pellets. HeLa and HCT-116 cancerous cell lines were used as positive controls. For sample preparation, cell lysis was performed using 20 µL of lysis buffer (supplemented with PMSF and β-mercaptoethanol) per 10^6^ cells before a 30 min incubation phase on ice. The samples were centrifuged at 12,000× *g* at 4 °C for 20 min. The supernatants were transferred to new Eppendorf tubes. For telomerase activity detection, 0.5 µL of sample, 4 µL of 5× telomerase reaction buffer, and 15.5 µL of nuclease-free water were mixed and incubated at 37 °C for 3 h. The reaction was quenched by heating the samples at 85 °C for 10 min. The samples were centrifuged at 1500× *g* for 10 s and stored on ice. The qPCR reactions were prepared in triplicate by mixing 1 µL of the prepared sample, 2 µL of primers, 10 µL of TaqGreen qPCR master mix, and 7 µL of nuclease-free water. The qPCR plates were sealed and centrifuged at 1500× *g* at ambient temperature for 15 s. The qPCR run conditions included an initial denaturation step of 10 min at 95 °C and 36 amplification cycles (i.e., denaturation over 20 s at 95 °C, annealing for over 20 s at 52 °C, and extension for over 45 s at 72 °C). Samples with a cycle threshold (Ct) > 33 were assessed as negative. The relative telomerase activity quantification between two samples was based on the 2^–ΔCt^ calculation method.

#### 2.6.5. Allogeneic FE002 Cellular Active Substance Qualification: Soft Agarose Colony Formation Assay for In Vitro Semi-Quantitative Tumorigenicity Assessment

A standard soft agarose cell colony formation assay (cell transformation assay) was used to assess the potential of FE002 primary chondroprogenitors to proliferate in non-adherent settings. The assays were performed in 24-well microplates. The solid agarose layer (i.e., the bottom layer) was composed of 0.6% agarose in PBS- and FBS-supplemented growth medium with 1% penicillin-streptomycin. The soft agarose layer (i.e., the top layer) was composed of 0.4% agarose and contained the investigated cellular materials (i.e., 125 viable cells/well to 10^4^ viable cells/well). Both agarose layers were sequentially prepared and of equal volumes. The tested cellular samples were FE002 primary chondroprogenitors that had been freshly harvested from confluent cultures. Positive control samples contained HeLa cancerous cells freshly harvested from confluent cultures. FBS-supplemented growth medium with 1% penicillin-streptomycin was added on top of the soft agarose layer, and the assay plates were incubated at 37 °C under 5% CO_2_ in a humidified incubator for 21 days. The plates were regularly microscopically assessed, and representative imaging was performed to comparatively assess the formation of non-adherent cell colonies.

### 2.7. Cytotherapeutic Finished Product Manufacturing Process: Chondrogenic Induction of Cell-Bearing Chondro-Gide Constructs

Autologous and allogeneic cellular active substance lots were used to seed Chondro-Gide scaffolds, which were incubated under chemical chondrogenic induction (i.e., two weeks of incubation). Technical specificities characterized each protocol (e.g., cellular active substance, chondrogenic induction medium composition). Overall, most of the parameters and technical specifications of the autologous and allogeneic protocols were similar, and any specific variations or differences were analyzed according to quality, functional attributes, and risk viewpoints. While maximal finished product dimensions correspond to 20 cm^2^ (i.e., 4 cm × 5 cm Chondro-Gide), smaller finished product units were experimentally investigated for sparing material use.

#### 2.7.1. Autologous Chondrocyte-Bearing Graft Manufacturing Process

Autologous chondrocytes were expanded in vitro before scaffold seeding. Chondro-Gide subunits with 1 cm × 1 cm dimensions were placed rough side up in 12-well plates and soaked with HPL for 1 h. Then, the seeding cell suspension (i.e., autologous cellular active substance in cell proliferation medium, passages 3–4, final cellular concentration of 2 × 10^6^ cells/cm^2^ scaffold) was homogeneously dispensed on the scaffold. The plates were incubated overnight at 37 °C under 5% CO_2_ in a humidified incubator. Residual seeding cell suspension was used for cell recovery controls. Then, volumes of 1 mL of autologous chondrogenic medium (i.e., DMEM–HAM’s F12 [1:1]; HPL 10%; L-glutamine; ascorbic acid 0.025 mg/mL; TGF-β1 10 ng/mL; ITS 1×; dexamethasone 10^−7^ M) were dispensed in each well, and the plates were incubated again. The cell-seeded scaffolds were maintained under chondrogenic induction for 16 ± 4 days, and medium exchanges were performed twice weekly. The constructs were finally rinsed thrice via immersion in warm PBS and were made available for further in vitro studies. The described processes were approved by the relevant health authorities in the framework of a clinical trial (i.e., NCT05651997).

#### 2.7.2. Allogeneic FE002 Chondroprogenitor-Bearing Graft Manufacturing Process

Cryopreserved FE002 primary chondroprogenitors were thawed and directly used for scaffold seeding. Chondro-Gide subunits (0.5–1 cm × 1 cm dimensions) were placed rough side up in 12-well plates, and the seeding cell suspension (i.e., allogeneic cellular active substance in cell proliferation medium, passage 6, final cellular concentration of 2 × 10^6^ cells/cm^2^ scaffold) was homogeneously dispensed on the scaffold. The plates were incubated overnight at 37 °C under 5% CO_2_ in a humidified incubator. Residual seeding cell suspension was used for cell recovery controls. The cell-seeded constructs were covered with 1 mL of allogeneic chondrogenic medium (i.e., high-glucose DMEM; 2 mM L-glutamine; ITS 1×; 10 nM dexamethasone; 10 ng/mL TGF-β3; 82 µg/mL VitCp). The cell-seeded scaffolds were maintained under chondrogenic induction for 15–18 days, and medium exchanges were performed thrice weekly. Macroscopic evaluation of the constructs was regularly performed over the course of the chondrogenic incubation phase. The constructs were finally rinsed thrice via immersion in warm PBS and were made available for further in vitro studies. To investigate the impact of scaffold size on the manufacturing process and endpoint functional attributes of the allogeneic finished product, 5 cm^2^ Chondro-Gide subunits were subsequently used for process validation.

### 2.8. Cytotherapeutic Finished Product Controls: Functional Validation

To comparatively assess the quality- and functionality-related attributes of the finished products (i.e., the autologous and allogeneic grafts), several in-process controls (IPC) and post-process controls (PPC) were performed. Specifically, cellular distribution throughout the constructs and cellular metabolic activity maintenance were assessed. Specific functionality parameters such as chondrogenic gene induction in 3D and ECM synthesis/deposition throughout the constructs were also assessed.

#### 2.8.1. MTT Staining for Assessing Metabolic Activity and Cell Distribution throughout the Constructs

An MTT assay was used to assess the endpoint cellular metabolic activity and cellular distribution throughout the Chondro-Gide scaffolds after incubation. Specifically, the MTT assay was used to confirm the adherence of the cells to the scaffold, the maintenance of cellular metabolic activity within the scaffold, and the quality of cellular colonization of the scaffold (i.e., the homogeneous repartition of the cells on the available surfaces). For endpoint analysis, the constructs were incubated at 37 °C for 2 h in a 5 mg/mL MTT solution. Following the rinsing of the constructs, photographic imaging was performed.

#### 2.8.2. Evaluation of Chondrogenic Gene Induction in the Constructs via RT-PCR

At various timepoints of the in vitro chondrogenic induction phase, the constructs were harvested and frozen at −80 °C for subsequent RNA extraction and gene expression analysis. The constructs were then mechanically disrupted in liquid nitrogen. The resulting powder was transferred to Eppendorf tubes containing TRIzol, and RNA extraction was performed according to the manufacturer’s protocol. RNA purity and concentration were quantified via spectrophotometry. Reverse transcription into cDNA was performed using 1 µg of RNA in a final volume of 20 μL using a PrimeScript RT reagent kit according to the manufacturer’s protocol. The reverse transcription cycle conditions were as follows: 37 °C for 15 min and 85 °C for 5 s. A real-time polymerase chain reaction (RT-PCR) was then performed in 96-well microplates. The reaction was performed using 1 μL of cDNA for a final volume of 20 μL using the KAPA SYBR Fast according to the manufacturer’s protocol. Fluorescence was acquired using the following cycling conditions: 95 °C for 3 min (i.e., enzyme activation) and 40 amplification cycles (i.e., 95 °C for 3 s and annealing extension at 60 °C for 30 s). Each sample was run in triplicate, and the relative expression level for each gene was normalized to GAPDH. Gene expression levels (for *Sox9*, *COL2*, and *ACAN*) were quantified using the 2^−∆∆Ct^ calculation method, as described elsewhere [66].

#### 2.8.3. DMMB Quantification for Assessment of ECM Synthesis and Deposition throughout the Constructs

The quantification of total glycosaminoglycans (GAG) in the cell-seeded constructs was performed using a DMMB kit according to the manufacturer’s protocol. Briefly, the constructs were harvested, washed once with PBS, weighed, and stored at −80 °C until further use. For the analysis, the samples were cut into small fragments with a scalpel. The fragments were transferred into Eppendorf tubes, and 1 mL of papain digestion buffer was added to each sample. Incubation was performed overnight at 65 °C for complete sample digestion, and the tubes were centrifuged at 10,000 rpm for 10 min. The supernatants were transferred into new Eppendorf tubes. Samples and analytical standard were diluted and mixed with 1 mL of dye reagent before incubation for 30 min at ambient temperature under gentle mechanical agitation. The samples were then centrifuged at 12,000 rpm for 10 min and carefully inverted to discard all the supernatant without disturbing the GAG pellet. Volumes of 0.5 mL of dissociation reagent were added on top of the pellets, and the samples were incubated for 10 min at ambient temperature with regular vortexing. Following the complete dissociation of the dye from the GAG pellet, the samples were centrifuged for 5 min at 12,000 rpm. Finally, volumes of 100 µL of standard or sample were transferred into 96-well microtitration plates, and the absorbance was determined at a wavelength of 656 nm. The relative GAG contents were determined with reference to the net weight of the constructs following harvest.

#### 2.8.4. Staining and Immunohistology for Specific ECM Component Visualization in the Constructs

At various timepoints of the in vitro chondrogenic induction phase, the constructs were harvested and fixed overnight at 4 °C in a 4% formalin solution, rinsed thrice with PBS, and transferred in 70% EtOH at 4 °C until inclusion in paraffin. After methyl methacrylate inclusion, thin 5-μm sections were cut and placed onto microscope slides, deparaffinized, and stained for over 30 min for specific ECM component visualization. The direct staining types were hematoxylin and eosin (H&E) and Alcian Blue (AB). Thereafter, the prepared immunohistology slides were processed using antibodies against specific ECM components, namely aggrecan (ACAN; Invitrogen primary antibody, N°AHP0022) and collagen I (COL1; Abcam antibody, N°ab138492). Following final revelation, the slides were microscopically assessed and imaged.

### 2.9. Translational Qualification of the Allogeneic Cytotherapeutic Finished Products

In order to further qualify the allogeneic cytotherapeutic finished products from a translational viewpoint, several assays were performed to validate the product validity period and its physical applicability for surgical suturing.

#### 2.9.1. Qualification of Allogeneic Finished Product Transport Medium and Product Validity Period Validation

Following manufacture and before surgical implantation, the finished product must be harvested, conditioned, and transported from the production suite to the clinical center. The transport medium for the autologous finished product was specified as normal saline with 20% autologous human serum (AHS) for a validity period of 6 h post-manufacture. For the allogeneic finished product, several synthetic conditioning and transport media were assessed to avoid the use of AHS and the related blood draw/blood product processing steps. Allogeneic finished product stability was experimentally investigated at ambient temperature over a time-period of 6 h using a diversified readout panel (i.e., grading of quality and functionality attributes). Specifically, 3 distinct conditioning and transport media were evaluated. The allogeneic finished products were produced over a period of 15 days, as previously described. Following harvest and rinsing, the constructs were transferred into transport tubes containing either (i) normal saline, NaCl 0.9%, (ii) PBS with 0.25% sodium hyaluronate, molecular weight range 1.0–1.25 MDa, or (iii) Opti-MEM medium without phenol red. The control samples were maintained under incubation in chondrogenic medium, while the other samples were submitted to a standardized transport protocol of 45 min over 12 km, followed by static storage (i.e., total transport and storage time-period of 6 h; all phases performed at controlled ambient temperature). All samples were finally harvested and controlled via MTT staining, total GAG quantification, and immunohistology.

#### 2.9.2. Validation of Allogeneic Finished Product Implantability via Suture Testing

The Chondro-Gide scaffold (with or without cells) can be implanted using fibrin glue and/or sutures depending on the retained clinical protocol. To verify that the allogeneic finished product possessed the appropriate structural/physical attributes for suture-based surgical implantation in the knee (i.e., the maintenance of physical integrity upon transport, handling, and suturing), an endpoint suture test was performed. After a manufacturing period of 15 days, the finished product samples were submitted to the standardized transport and storage protocol. Then, a standard knot was tied in each sample using a Monosyn 6/0 suture kit, and a gentle mechanical challenge was applied to the finished products (i.e., simulation of finished product handling and surgical knot tightening). An endpoint MTT assay was performed before and after suturing, and imaging was performed.

### 2.10. Statistical Analysis and Data Presentation

To statistically compare the average values from the two datasets, a paired Student’s *t*-test was applied. The normality of data distribution was appropriately validated prior to the application of parametric tests. A *p*-value < 0.05 was used to determine statistical significance. Discrete data are presented using histograms and box-and-whisker plots, while continuous data are presented using broken-line graphs. Calculations and data presentation were carried out using Microsoft Excel, Microsoft PowerPoint (Microsoft Corporation, Redmond, WA, USA), and GraphPad Prism version 8.0.2 (GraphPad Software, San Diego, CA, USA).

## 3. Results

### 3.1. FE002 Primary Chondroprogenitors Possess Quality and Safety Attributes Compatible with Clinical Tissue Engineering

Within existing clinical ACI applications, the use of HPL as a cell proliferation medium supplement has been implemented successfully following functional validation against the historically used FBS [66]. Therefore, our experimental results initially confirmed that 5% HPL could technically be used as an alternative to 10% FBS for the monolayer expansion of FE002 primary chondroprogenitors (Figures S1 and S2). However, based on the existing body of research on clinical-grade FE002 primary chondroprogenitors, the substitution of FBS by HPL has not yet been undertaken (mainly for stability reasons), and all further data presented herein were gathered with FBS-cultured cells.

From a quality point of view, the proteomic characterization of the allogeneic FE002 cellular active substance yielded some insights into the potential molecular contributions underlaying the intended therapeutic effects of the cells (Table S1). Specifically, cell therapies are postulated to partly act by means of paracrine effects, a concept which directed the analyses toward the soluble fraction of FE002 primary chondroprogenitor lysates. These were found to mainly contain (in relatively high abundance) MMPs/TIMPs, growth factors, and cytokines (Table S1). Notably, MMP-2, MMP-13, TIMP-1, and TIMP-2 were identified in concentrations > 30 ng/mg of total proteins (Table S1). MMPs/TIMPs are conjointly involved in the processes of ECM regulation, which is of high functional relevance in chondral defects [67]. The growth factor FGF-2 is known to stimulate chondrocyte or mesenchymal stem cell proliferation and plays a positive role in cartilage healing [67]. The growth factor HGF has been shown to induce in vitro rodent chondrocyte proliferation and ECM synthesis [68]. Another identified protein, sgp130, is a negative regulator of IL-6, with the latter being involved in cartilage degradation (Table S1). Similarly, sTNFRI and IL-1Ra are negative inhibitors of TNF and IL-1 signaling (i.e., they are proinflammatory, negatively impacting cartilage), respectively. Overall, FE002 primary chondroprogenitors were found to contain potentially useful proteinic components for orthopedic cytotherapeutic applications (Table S1).

From an efficacy point of view, it was firstly confirmed that FE002 primary chondroprogenitors could not yet reach senescence at passage levels equal or superior to that of the cellular active substance lots (i.e., the maintenance of physiological activity at passages 7–8, Figure S3). Furthermore, from a preliminary safety viewpoint, the small amounts of isolated senescent cells (i.e., 1.5–3.0% of total cells) that were recorded confirmed that FE002 primary chondroprogenitors are not immortal and therefore present low potential for uncontrolled and indefinite proliferation (Figure S3). Secondly, specific in vitro safety characterization of the allogeneic cellular active substance was performed to complement and support existing in vivo safety data. In the soft agarose assay, significant non-adherent colonies of HeLa cells were rapidly observed, and they continued to grow over the 21-day incubation phase (Figure S4). Conversely, no anchorage-independent cellular proliferation or tumoral growth-like activity was recorded for the FE002 progenitor cell group (Figure S4). In the telomerase activity assay, the FE002 cells (i.e., clinically usable passage levels) were found to possess low telomerase activities (i.e., at the lower limit of detection of the test) that were comparable in value to those of seven patient primary HAC cell types (i.e., previously safely clinically used for ACI, Figure S5). Conversely, both positive controls (i.e., HeLa and HCT-116 cancerous cells) were found to possess high telomerase activity (as expected), thereby confirming the validity of the experimental setup (Figure S5). Overall, no safety-related concerns were evidenced in vitro for the FE002 primary chondroprogenitors (congruent with and complementary to existing safety data, including a large animal GLP study), further confirming their applicability in subsequent translational applications.

### 3.2. FE002 Primary Chondroprogenitors Possess Quality and Functionality Attributes Compatible with the Controlled GMP Manufacturing of Chondrogenic Cellular Active Substances

**Figure 1:**
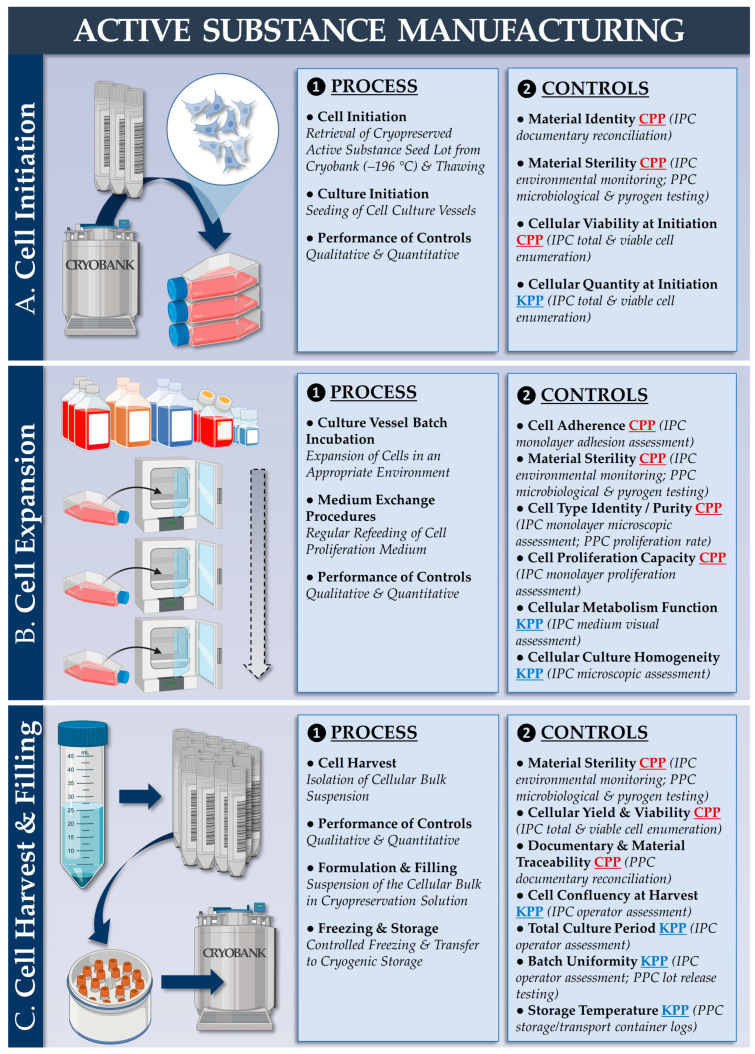
Schematic presentation of the manufacturing and control processes for the autologous or allogeneic chondrogenic cellular active substances. The process describes a primary cell amplification and cryopreservation cycle, starting with master cell bank (MCB) or working cell bank (WCB) vials of HACs or FE002 primary chondroprogenitors. (**A**) Cellular seeding material initiation from cryopreservation with rapid thawing and culture vessel seeding. (**B**) In vitro cellular expansion in monolayers for cellular active substance lot manufacture. (**C**) Cellular bulk harvest and cellular active substance lot processing for cryopreservation. CPP, critical process parameter; HAC, human articular chondrocytes; IPC, in-process control; KPP, key process parameter; MCB, master cell bank; PPC, post-process control; WCB, working cell bank.

Based on the existing research on clinical-grade primary HAC cell type establishment and GMP manufacturing, the stability of the FE002 primary chondroprogenitors was firstly assessed following 3 years of cryostorage. Following parallel in vitro initiation with primary HACs, it was confirmed that the FE002 cells possessed appropriate key and critical quality attributes (e.g., adherence, morphology, proliferation) in recovery cultures after storage (Figure S6). Based on this validation of the cryopreservation phase, a parametrically defined and controlled manufacturing process was devised for the chondrogenic cellular active substances (designed to be applicable to both autologous HACs and allogeneic FE002 primary chondroprogenitors, Figure 1).

**Figure 2:**
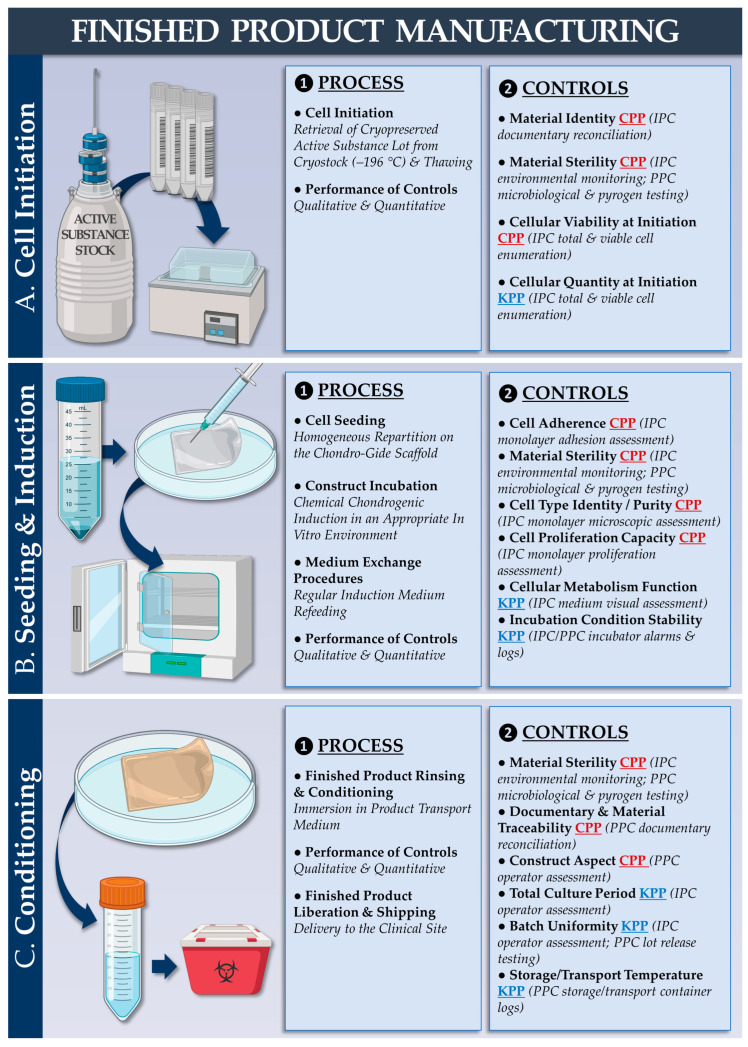
Schematic presentation of the manufacturing and control processes for the allogeneic finished cytotherapeutic product (i.e., Chondro-Gide scaffolds bearing FE002 primary chondroprogenitors). The process describes cellular active substance lot initiation, scaffold seeding and chondrogenic induction, and finished product conditioning for transport. (**A**) Cellular active substance initiation from cryopreservation with rapid thawing. It is important to note that, in the autologous protocol, an additional in vitro HAC monolayer expansion phase is carried out at this point. (**B**) Seeding of the cellular active substance on the Chondro-Gide scaffold and incubation of the constructs under chemical chondrogenic induction. (**C**) Endpoint harvest of the finished cytotherapeutic product lot and conditioning for transport to the clinical site. CPP, critical process parameter; HAC, human articular chondrocytes; IPC, in-process control; KPP, key process parameter; PPC, post-process control.

The corresponding quality attributes (i.e., active substance) are presented in Table S2. From a technical standpoint, the most significant difference between the autologous and allogeneic workflows is the possibility to serially manufacture extensive allogeneic cell lots, contrasting with patient-specific autologous HAC manufacturing campaigns. Regarding finished product manufacturing, a second parametrically defined and controlled manufacturing process was devised (designed to be applicable to autologous and allogeneic protocols) based on the approved autologous protocol (Figure 2).

The corresponding quality attributes (i.e., finished products) are presented in Table S3. Illustrative experimental records from the autologous and allogeneic finished product manufacturing campaigns are presented in Figure S7a,b (i.e., upscaling studies using 5 cm^2^ Chondro-Gide subunits), respectively. For simplified manufacturing process benchmarking (i.e., autologous versus allogeneic protocol), a list of the production process parameters (i.e., cellular active substance and finished product manufacturing) is presented in Table 1.

An illustrated workflow describing the temporal constraints and the risks associated with the autologous and/or the allogeneic protocol is presented in Figure S8. Generally, the two processes were considered to be technically overlapping. The main differences in the allogeneic process were the use of FBS instead of HPL for cell expansions, the direct use of cryopreserved cellular active substance for Chondro-Gide seeding, and the tailoring of the chondrogenic induction medium for optimal ECM deposition (Table 1).

### 3.3. FE002 Primary Chondroprogenitors Possess Functional Attributes Comparable to Those of Clinical-grade HACs within Chondrogenically Induced Chondro-Gide Constructs

To maximize the therapeutic potential of the intervention, the presence of viable and functional cells at the time of construct implantation in the knee is required. Endpoint MTT assays were performed on the autologous and allogeneic finished products, initially confirming that the cells homogeneously colonized the Chondro-Gide scaffolds and retained significant metabolic activity (Figure S7(aE,bF2)). Furthermore, the cells colonized one of the two scaffold layers (i.e., rough side), as expected (Figure S7b).

**Figure 3:**
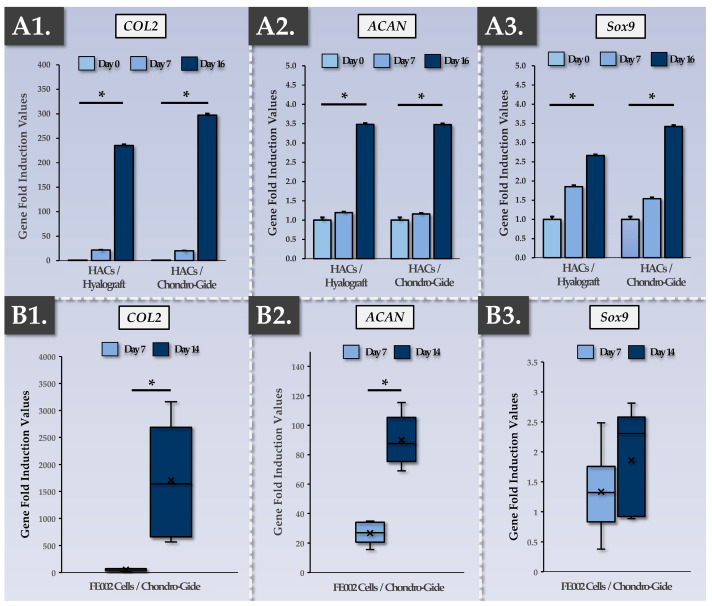
Functional characterization of the autologous and allogeneic finished products, as assessed according to their evolutive chondrogenic gene expression levels during construct incubation. (**A1**–**A3**) Relative chondrogenic gene (i.e., *COL2*, *ACAN*, *Sox9*) fold induction values at various timepoints of autologous finished product incubation (assessed for Hyalograft and Chondro-Gide scaffolds, respectively). Both scaffolds were assessed as being functionally equivalent, and endpoint chondrogenic gene expression was highly significantly increased compared to the baseline in all groups (*p*-values < 0.01). (**B1**–**B3**) Relative chondrogenic gene (i.e., *COL2*, *ACAN*, *Sox9*) fold induction values at various timepoints of allogeneic finished product incubation (assessed for Chondro-Gide scaffolds). Endpoint chondrogenic gene expression was highly significantly increased for *COL2* (*p*-value < 0.01) and *ACAN* (*p*-value < 0.0001). Furthermore, endpoint chondrogenic gene expression was significantly higher in value for *COL2* and *ACAN* (*p*-values < 0.01) compared to the respective endpoint induction levels of the same genes in the autologous finished products (i.e., the Chondro-Gide groups). Experimental replicates (n ≥ 3) and repetitions were used for the assays. Statistically significant differences are marked by an asterisk (i.e., “*”). ACAN, aggrecan; COL, collagen.

Regarding specific chondrogenic function in the constructs, the cultured cells must be able to readopt a chondrogenic phenotype after monolayer expansion (i.e., where transient de-differentiation occurs). The results of finished product functional characterization at the gene expression level showed that, during the in vitro 3D chondrogenic induction phase, specific genes of interest were induced in HACs and in FE002 primary chondroprogenitors (Figure 3).

Specifically, previous internal research showed that HAC chondrogenic genes (*COL2*, *ACAN*, *Sox9*) were induced following the chemical induction of 3D cell pellets [66]. In the present study, our results showed that similar functions could be obtained upon placing HACs within two types of implantable sheet scaffolds, with highly significant chondrogenic gene induction taking place at the 16-day timepoint (Figure 3A). As the Chondro-Gide scaffold was already clinically implemented for the second-generation ACI protocol (i.e., NCT04296487) and retained for the MACT protocol (i.e., NCT05651997), it was used for the functional qualification of the FE002 primary chondroprogenitors as well (Figure 3B). Notably, regarding the allogeneic cell group, *Sox9* expression was constitutively high and was not significantly (*p*-value = 0.296) induced following the construct incubation phase (Figure 3(B3)). However, extremely potent *COL2* and *ACAN* induction were recorded in the FE002 groups, with endpoint values over 10× higher than those of the autologous HAC-based constructs (Figure 3(B1,B2)). Overall, it was shown that the autologous and allogeneic cells were capable of re-expressing specific chondrogenic genes following appropriate chemical induction in 3D, with quantitative advantages favoring the allogeneic FE002 cells (Figure 3). Additionally, it was shown that a two-week chondrogenic induction period resulted in the exponential induction of *COL2* and *ACAN* genes and that, from a functional standpoint, a two-week chondrogenic induction period is preferrable over a 7-day induction period (Figure 3).

**Figure 4:**
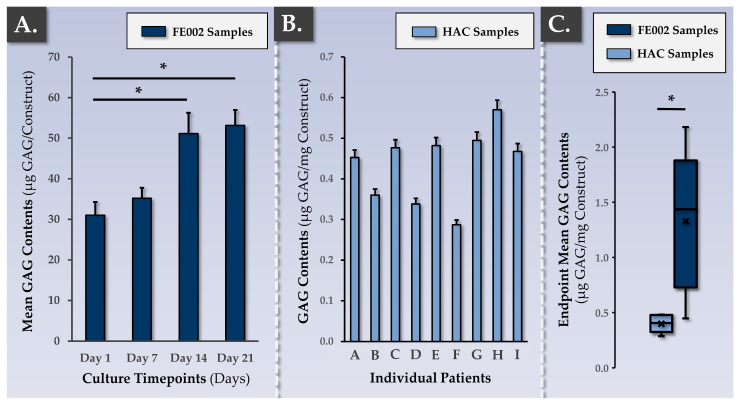
Functional characterization of the autologous and allogeneic finished products, as assessed via total GAG quantification within the constructs. (**A**) Increase in GAG contents over time within constructs bearing allogeneic FE002 primary chondroprogenitors. Experimental replicates (n ≥ 6) and repetitions were used for the assay. Statistically significant differences (*p*-values < 0.05) are marked by an asterisk (i.e., “*”). (**B**) Interpatient variability in terms of endpoint GAG contents within freshly harvested autologous constructs. Experimental replicates (n ≥ 3) from nine different donors were used for the assay. (**C**) Comparison of endpoint GAG contents between constructs bearing HACs (i.e., 16 days of induction) and constructs bearing FE002 primary chondroprogenitors (i.e., 14 days of induction). Experimental replicates (n ≥ 6) and repetitions were used for the assay. Statistically significant differences (*p*-value < 0.05) are marked by an asterisk (i.e., “*”). GAG, glycosaminoglycan; HAC, human articular chondrocytes.

To validate the functional advantages generated by the chondrogenic induction phase and measured via gene expression analysis, finished product functional characterization was performed at the protein level, focusing on ECM components. A DMMB assay for total GAG quantification was employed in various experimental setups (Figure 4).

In the allogeneic FE002 cell group, a time-course of GAG deposition in the Chondro-Gide scaffold showed a significant (*p*-value < 0.05) increase between the 7-day and 14-day timepoints but no further rise at the 21-day timepoint (Figure 4A). Such results were congruent with the data gathered at the gene expression level and validated the chondrogenic induction phase duration of approximatively two weeks (Figure 3). Then, the DMMB assay was used to assess the interpatient variability in endpoint total GAG contents within the autologous finished product (Figure 4B). Furthermore, mean endpoint total GAG contents were compared between the autologous and allogeneic groups, wherein the FE002 cells were found to deposit much more ECM throughout the constructs than the HACs (Figure 4C).

**Figure 5:**
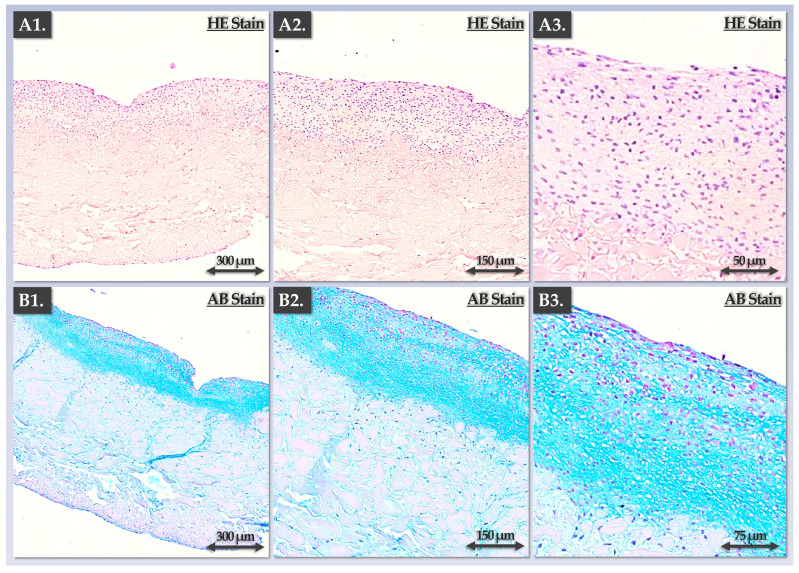
Endpoint functional characterization of the autologous finished product (as assessed by immunohistology for constructs bearing HACs). (**A1**–**A3**) Sections of a construct following HE staining. (**B1**–**B3**) Sections of a construct following AB staining. The results showed the zone-specific localization of the HACs (i.e., rounded cells within lacunae) and the deposited ECM (i.e., one defined construct zone), as expected. AB, Alcian Blue; ECM, extracellular matrix; HAC, human articular chondrocytes; HE, hematoxylin and eosin.

For a qualitatively enhanced investigation of the ECM deposition activities in the Chondro-Gide constructs by HACs and FE002 primary chondroprogenitors, several histology assays were performed. Firstly, endpoint cellular localization and ECM organization were visualized in the autologous constructs, showing appropriate structural and composition attributes (Figure 5).

**Figure 6:**
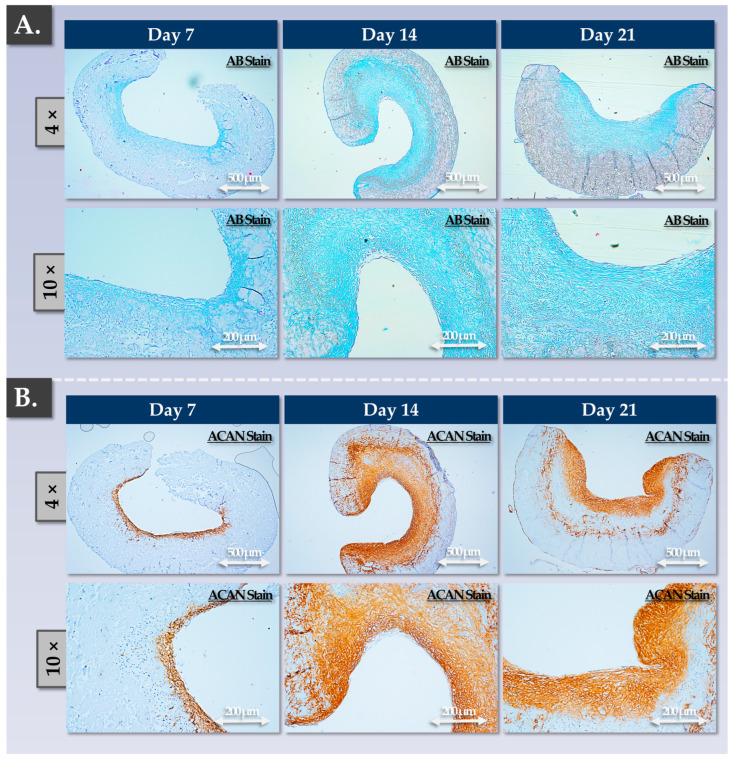
Functional characterization (time-course) of the allogeneic finished product, as assessed via immunohistology for constructs bearing FE002 primary chondroprogenitors. (**A**) Construct sections at various timepoints of the incubation phase following AB staining. (**B**) Construct sections at various timepoints of the incubation phase following ACAN staining. Overall, the results showed that significant ECM deposition occurred between days 7 and 14 of the construct incubation phase. AB, Alcian blue; ACAN, aggrecan; ECM, extracellular matrix.

As expected, cellular presence and ECM deposition were concentrated within one layer of the scaffold (the rough side; Figure 5). Furthermore, cellular morphology was observed to be rounded, and the cells were embedded in the lacunae (Figure 5(A3,B3)). Regarding the allogeneic grafts, progressive ECM deposition was observed in a time-course immunohistology setup (Figure 6). Therein, the experimental results were congruent with the gene expression and GAG quantification readouts, showing a strong increase in ECM deposition between the 7-day and the 14-day timepoints (Figure 6). Furthermore, no significant increase in ECM content was evidenced at the 21-day timepoint, and the homogeneity in the aggrecan deposition was lower than that at the 14-day timepoint (Figure 6).

**Figure 7:**
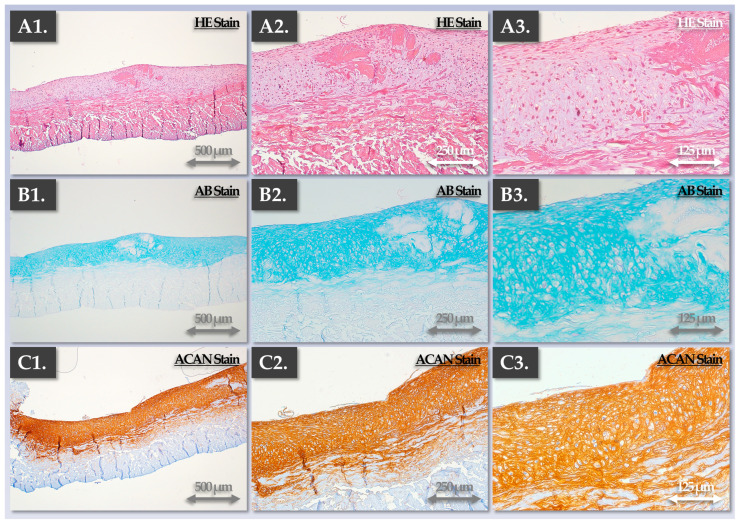
Endpoint characterization of the allogeneic finished products (as assessed via immunohistology for constructs bearing FE002 primary chondroprogenitors). In addition to significant ECM deposition in one layer of the constructs, the cells were observed to be rounded and localized in the lacunae, as expected. (**A1**–**A3**) Hematoxylin and eosin staining. (**B1**–**B3**) Alcian Blue staining. (**C1**–**C3**) Aggrecan staining. AB, Alcian Blue; ACAN, aggrecan; ECM, extracellular matrix; HE, hematoxylin and eosin.

Subjecting the allogeneic grafts to multiparametric endpoint histological evaluation enabled us to confirm that appropriate structural and qualitative attributes were obtained and were comparable to those of the autologous grafts following two weeks of chondrogenic induction (Figure 5 and Figure 7).

The various control parameters, methods, and acceptance criteria used for the parallel functional qualification of the autologous and allogeneic constructs are presented in Table 2, along with the corresponding experimental assessments.

A summary of the various relevant finished product attributes (classified by control parameter type) is presented in Table 3.

Specifically, each control parameter subtype was identified with respect to its use in the finished product manufacturing process itself (i.e., IPC vs. PPC), in its development/validation, and in its implementation (i.e., release criterion, Table 3). Overall, the gathered experimental data confirmed that the produced allogeneic grafts were qualitatively non-inferior or functionally superior to the autologous grafts, with the latter being approved for clinical investigational use (i.e., NCT05651997). Multi-parametric functional data confirmed and validated that a two-week chondrogenic induction period was most appropriate for the considered finished products (Figure 3, Figure 4, Figure 5, Figure 6 and Figure 7). Furthermore, no differences were observed between the 1 cm^2^ and 5 cm^2^ allogeneic constructs in terms of endpoint functional attributes, confirming that the described manufacturing protocol is readily upscalable (Figure S7).

### 3.4. Allogeneic Finished Products Possess an Appropriate Pharmaceutical Form and Stability Attributes for Clinical Orthopedic Bioengineering

**Figure 8:**
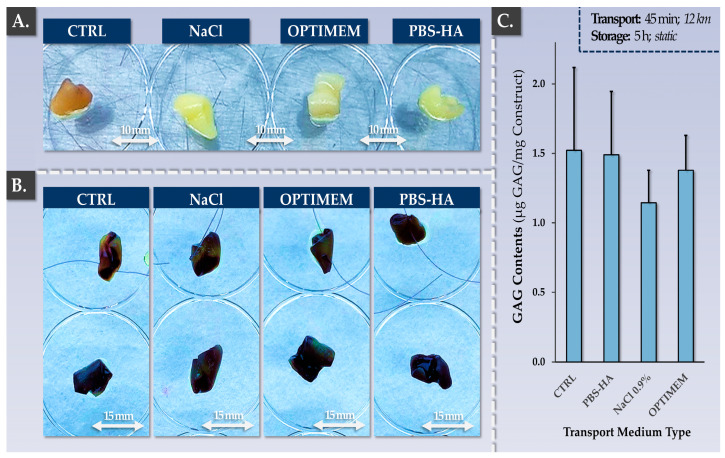
Validation results for allogeneic finished product transport medium and construct suturability. (**A**) Freshly harvested finished products before conditioning for transport/storage. Experimental replicates (n = 3) were used for the assay. (**B**) MTT-stained finished products after the application of the standardized transport protocol. Constructs presented in the top row were additionally submitted to the suture test before MTT staining. Experimental replicates (n = 3) were used for the assay. (**C**) Impact of the transport protocol on the total GAG contents of the constructs. Experimental replicates (n = 6) were used for the assay. CTRL, control; HA, hyaluronic acid; PBS, phosphate-buffered saline.

In order to confirm that the allogeneic finished products maintained appropriate quality and functionality attributes at least up until surgical implantation in the knee, specific validation studies were performed. Firstly, it was confirmed that ambient temperature storage and transport for a total time-period of 6 h did not significantly impact cellular colonization and viability nor the total GAG contents of the constructs (Figure 8).

Similar results were obtained for the three types of tested transport media (Figure 8). At the end of the manufacturing phase and following transport/storage, the constructs were evaluated via macroscopic assessment, cell viability assessment, total GAG quantification, and histology for three different transport media (Table 4).

While the GAG contents were found to be 25% lower on average in the NaCl group compared to the control group, no statistically significant difference was found (*p*-value = 0.370, Figure 8C). Macroscopically, no inexplicable or unusual changes in construct physical attributes were noted after harvest (Figure 8A). Structurally, no significant modifications were evidenced between the groups via our histological analyses (Table 4). Secondly, it was shown that the constructs could be handled and sutured using standard surgical threads (Figure S9). Specifically, it was shown that construct handling, suturing, and gentle mechanical challenging did not result in the disturbance of the biological materials present on the samples (Figure 8B, top row). Overall, it was experimentally confirmed that the retained manufacturing process and technical specifications enable the production of clinically usable allogeneic grafts of appropriate quality for orthopedic implantation.

## 4. Discussion

### 4.1. Progressive Translational Development of Cell Therapies for Cartilage Repair/Regeneration: Extensive Manufacturing Experience and Long-Term Clinical Research on ACI

Cartilage-oriented regenerative strategies are currently far from being fully satisfactory, and the search for effective disease-modifying interventions is ongoing [69,70,71]. In order to meet increasing clinical needs, important translational efforts are being allocated toward the optimization of existing ACI protocols or the creation of novel approaches [11,12,72,73,74,75,76]. Several generations of ACI have been clinically investigated and shown to yield positive impacts for treated patients [1,2,3,11,12,13,14,15]. Importantly, extensive, long-term, and large-scale clinical human data are available for ACI [15,22,29,30]. In one study, first-generation ACI involved the arthrotomic implantation of expanded HACs under a periosteal flap [7,14]. The aim of this study was to durably restore tissular structures and functions following lesion debridement and the creation of an optimal local environment for the cell-based product. Good clinical outcomes were obtained, yet a major cause of failure was the development of periosteal hypertrophy, requiring new surgical interventions [8,13,14]. Additionally, the surgery requires an open-joint procedure and the harvest of a periosteal flap, which is fragile and can tear during suturing.

In second-generation ACI, the periosteal flap was replaced by an artificial membrane (e.g., Chondro-Gide) [8,14]. This substitution improved outcomes, as the procedure was less invasive (no periosteal tissue harvest); less surgical complications were observed, and a reduction in hypertrophy development was recorded [2,8,11]. However, the procedure still required open surgery, which brings risks of complication. Furthermore, a risk of injected cell leakage or inhomogeneous repartition on the lesion surface exists. McCarthy et al. directly compared the clinical and histological outcomes between first- and second-generation ACI [38]. Patients implanted with Chondro-Gide membranes demonstrated a higher cellular morphology score (i.e., ICRS II score), a better surface morphology for treated medial femoral condyle defects, and a higher proportion of hyaline cartilage formation (i.e., OsScore) [38]. These results demonstrated that the use of Chondro-Gide membranes resulted in a better quality of tissular repair [38].

Overall, considering the available clinical data on the various generations of ACI led us to conclude that the interventions are generally safe and effective and that the successive technical updates in therapy/product manufacturing protocols have been clinically beneficial [14,15,16,17,77,78,79,80]. Specifically, it was shown in multiple settings and by various clinical groups that the use of the Chondro-Gide membrane was of high therapeutic utility in a variety of treatment strategies. The comprehensive consideration of the elements presented hereabove enabled the local implementation of the NCT05651997 clinical trial, which was based on the robust global track records of the Chondro-Gide membrane and third-generation ACI.

### 4.2. Safety, Quality, and Efficacy Attributes: FE002 Primary Chondroprogenitors Are Compatible with Modern Clinical Regenerative Medicine Requirements

Since the establishment of the cell source in 2009 under the Swiss progenitor cell transplantation program, FE002 primary chondroprogenitors have been exploited as clinical-grade cytotherapeutic materials [49,50]. Specifically, extensive technical work has validated the applicability of such cells in industrial-scale manufacturing workflows for transposition to GMP manufacturing [50]. Furthermore, previous preclinical research has shown the versatility and high potential of FE002 primary chondroprogenitors as promising contenders in cell-based orthopedic regenerative medicine [50,51,52,53,54,81]. Some of the advantages of using such an allogeneic cellular active substance for cartilage bioengineering involve the off-the-freezer availability of standardized biologicals, rationalized manufacturing workflows, and drastically reduced operative burdens [50].

However, major concerns regarding the development of novel cell-based protocols for human cytotherapeutic use are linked to biological material safety, especially in allogeneic contexts. Notably, FE002 primary chondroprogenitors have been implanted in vivo in a variety of xenogeneic settings (including a caprine GLP study of knee cartilage defects) [50,51,52]. Furthermore, the original experimental results presented in this study have served to complement and enhance the available body of knowledge on the safety attributes of FE002 primary chondroprogenitors (finite in vitro lifespan, low telomerase activity, no anchorage-independent cell growth). From a mechanistic viewpoint, the identification of the main soluble constituents of the FE002 allogeneic biological materials (e.g., growth factors, cytokines) has provided some insight into the possible biochemical cues at play in the paracrine modulation of pathological environments (Table S1).

From a translational viewpoint, our combined cell manufacturing and clinical cytotherapeutic experiences enabled us to tangibly set forward the established protocols and processes (i.e., autologous and allogeneic, Figure 1 and Figure 2). Regarding local GMP manufacturing and the clinical administration of HAC-based preparations, more than 67 patients have been treated to date (NCT04296487 clinical trial) [48]. Regarding GMP manufacturing and the clinical use of primary progenitor cells (e.g., FE002 primary dermal progenitor fibroblasts), more than 300 patients have been treated to date [65]. Overall, the appropriate consolidation of the locally available resources and research should enable the timely transposition of orthopedic protocols from an autologous to an allogeneic setting using FE002 primary chondroprogenitors.

### 4.3. FE002 Primary Chondroprogenitors Are Functionally Comparable to Clinical-grade HACs in Chondro-Gide Constructs

The functional characterization of FE002 primary chondroprogenitors has previously been reported by multiple research groups and in a variety of product prototypes [50,51,52,81]. The specific methodological advantage of the present study lied within the use of a regulatorily approved autologous approach and clinical-grade HAC materials as a baseline for manufacturing process benchmarking and parallel functional qualification. Therein, it was shown that FE002 primary chondroprogenitors equaled or outperformed patient primary HAC cell types in terms of function in the retained Chondro-Gide construct (Figure 3 and Figure 4). Specifically, it was shown that finished products with appropriate quality and functionality attributes could be obtained using both protocols, notwithstanding the specific technical adaptations (Table 1, Tables S2 and S3). The simultaneous consideration of both approaches enabled us to devise optimized manufacturing processes and related controls, which were validated as being applicable to the HAC-based and the FE002 primary chondroprogenitor-based protocols (Figure 1 and Figure 2, Table 2). While direct comparison between the two approaches was not possible (due to specific process adaptations), parallel functional qualification indicated that both finished product types conformed to the specified requirements (Table 2).

To strengthen the rationale of using Chondro-Gide scaffolds for therapeutic cell chondrogenic induction, an alternative autologous approach (i.e., N-TEC, engineered nasal cartilage) has been discussed [32,57,82]. Nasal chondrocyte-based tissue-engineered cartilage has been extensively studied at preclinical and clinical levels, garnering a robust scientific, technical, and clinical track record [32,57,73]. Specifically, chondrocytes isolated from nasal septa were cultured and chondrogenically induced on Chondro-Gide scaffolds for up to two weeks [32,73]. Favorable therapeutic effects have been shown for orthopedic patients following osteoarthritic knee cartilage defect management using the N-TEC protocol [32]. Notably, the methodological elements of the N-TEC protocol and its iterative technical updates were closely considered for devising the autologous and allogeneic workflows presented herein [66,73]. Therein, manufacturing technical specificities and stepwise control implementation from the N-TEC approach were considered as bases for the validation of both reported protocols (autologous and allogeneic) using the Chondro-Gide scaffold [73].

A notable technical divergence in this study between the presented approaches (autologous vs. allogeneic) was the use of HPL for the autologous protocol and the use of FBS in the allogeneic protocol (Table 1). While the implementation of HPL as a cell proliferation medium supplement may easily be justified from a risk reduction viewpoint, the long-term functional impact on expanded chondrogenic cells remains under investigation. Specifically, several studies have shown the functional equivalence of FBS and HPL for the in vitro manufacturing of chondrogenic cells [66,83,84,85,86,87,88,89]. Furthermore, the upscaling of cell manufacturing for ACI has technically excluded the use of AHS as a culture supplement due to limited available quantities [72,74]. Therefore, specific concerns have been voiced over the impact of HPL supplementation on the in vitro chondrogenic potential of the cellular active substance; however, the available reports are not all congruent, and the overall impact on the therapeutic efficacy of the intervention remains unknown [74]. Overall, while several new cell proliferation medium supplements are available and conform to the modern standards of animal material-free workflows, FBS remains the gold standard due to its proven manufacturing and clinical track record [84,85,86,87]. These elements were considered to justify the maintenance of FBS for the industrial manufacturing of FE002 primary chondroprogenitors, despite the evident technical applicability of HPL (Figures S1 and S2) [50].

### 4.4. Autologous versus Allogeneic Approaches to Large (Osteo)-Chondral Defects of the Knee: Comparative Burden Analysis for Clinical Pathway Rationalization

A main advantage of adopting an allogeneic cell-based approach to manage large knee (osteo)-chondral defects is the reduction in operative burdens and donor-site morbidity [50,90]. Specifically, allogeneic workflows normally require only one orthopedic intervention at the time of matured graft implantation in the joint (Figure S8). Furthermore, it was validated that AHS was not necessary in the finished product transport medium for the FE002 cell-based allogeneic constructs, removing the need for autologous blood draw (Figure 8, Table 4). Such process simplifications may be interpreted positively in the case of the presented allogeneic protocol and from multiple standpoints (clinical pathways, technical risks, resource allocation).

Regarding the clinical pathway rationalization with the use of the allogeneic protocol, numerous logistical advantages may be yielded by the off-the-freezer availability of the FE002 cellular active substance (Figure 1 and Figure S8) [50]. Specifically, the operative program may be devised around a single orthopedic intervention for graft implantation, and manufacturing activities may be retro-planned accordingly, without the time constraints linked to autologous biopsy processing (Figure S8) [48]. Therefore, the serial manufacturing of FE002 allogeneic cellular active substance lots allow for significant potential organizational gains at the institutional level.

Regarding the technical risk reduction in the allogeneic approach, the amount of process steps and the number of repetitions of said steps is determinant. Indeed, the autologous protocol requires primary patient HAC cell type establishment, HAC expansion, and finished product formulation steps to be performed for each new patient (Figure S8) [48]. Conversely, the exploitation of a standardized allogeneic cell source such as FE002 primary chondroprogenitors does not require renewed cell type establishment, and the same cellular active substance lot may be used quasi-universally for different patients [50]. Therein, standard FE002 cellular active substance manufacturing batches may potentially serve for the serial preparation of >20 allogeneic grafts (40 mm × 50 mm). Thus, all technical means that result in a reduction in manufacturing steps or repetitions may provide tangible reductions in biosafety-related risks or manufacturing failure-related risks (Figure S8). Importantly, the high inter-patient variability, which impacts autologous cellular active substance manufacturing activities, may be avoided in the allogeneic approach, which enhances process standardization [48]. At the finished product level, the original data presented in the present study showed the superior performance of the FE002 cells in terms of GAG synthesis but also showed higher variability compared to the HAC-based constructs (Figure 4C). Such results were explained by the different scale of chondrogenic gene expression under induction (i.e., >ten-fold higher expression in allogeneic constructs) and were not interpreted negatively due to the fact that only minimal requirements are specified within functional controls (Figure 3, Table 2).

Regarding the comparative analysis of resource allocation for the autologous and allogeneic approaches, the technical and clinical simplifications described hereabove lead to overall cost rationalization within the allogeneic approach (Table 1). Specifically, the sparing use of manufacturing resources may be achieved via serial cell batch production instead of patient-specific production (Figure S8). Furthermore, less surgeon-related and operating room-related resources are needed, as the autologous biopsy harvest procedure is abolished in the allogeneic protocol (Figure S8). Importantly, as the specific case of second-generation ACI is reimbursed as a lump sum by universal healthcare coverage in Switzerland, any means that can lower the overall cost of the cell-based orthopedic intervention may potentially demonstrably lead to higher specific public healthcare efficiency, provided that therapeutic outcomes are at least equivalent [48].

However, the various advantages presented hereabove in favor of the implementation of allogeneic cell-based protocols are counterbalanced by the time- and resource-consuming process of obtaining the ad hoc regulatory approvals [50,91,92,93,94,95,96,97,98,99]. Specifically, extensive risk-based approaches to the transition from autologous to allogeneic investigational medicinal products (IMP) must be followed, along with appropriate (i.e., specific and general) risk analyses (for product quality, purity, efficacy, safety, and stability; see Tables S4–S7) [48]. The tangible consideration of FE002 allogeneic cellular active substance substitution into an existing technical and clinical workflow (autologous to allogeneic transposition) is highly advisable compared to de novo process development and implementation.

### 4.5. Identified Study Limitations and Future Clinical Research Directions

The main technical limitations of the presented study were related to the use of specific manufacturing processes for the autologous and allogeneic materials, which did not enable a strict comparison of both approaches. Importantly, both manufacturing processes were developed and optimized, respectively, around HACs and FE002 primary chondroprogenitors to maximize cellular active substance and finished product quality and functionality attributes [48,49,50,66,100,101]. Therefore, we chose to parallelly qualify the two types of finished products using the respective technical specifications of both protocols (i.e., those regulatorily approved for the autologous protocol and those used for the industrial manufacturing of the allogeneic grafts). This was preferred to using an identical manufacturing process for the autologous and allogeneic cells, which would have enabled the strict functional benchmarking of the materials. This option was not favored, as the relevance of appropriate functional attribute development is generally higher than that of process technical specificities (all risks being equal and mitigated). By extension, the strict quantitative benchmarking of both approaches is probably of low translational relevance as, for such similarly behaving biologicals (i.e., in terms of endpoint functional attributes), it is unclear if superior in vitro performance would correspond to differential therapeutic benefits for the patients [102,103,104,105].

Regarding the scale of the presented work (i.e., Chondro-Gide membrane sub-units), further validation studies are warranted in order to generate constructs of appropriate dimensions for the management of large knee (osteo)-chondral defects (>10 cm^2^). When working with qualified scaffold lots, high robustness in finished product manufacturing processes was recorded, and our experimental results regarding manufacturing protocol upscaling confirmed endpoint functional equivalence at two different size scales (1 cm^2^ vs. 5 cm^2^). Therefore, the use of full 20 cm^2^ Chondro-Gide membranes would require the adaptation of incubation vessels and contact–process consumables for ease of processing in GMP manufacturing environments.

As previously mentioned, a large body of scientific and technical research regarding the clinical-grade allogeneic FE002 primary chondroprogenitor cell source is currently available. Notably, the in vitro and in vivo safety of the FE002 cells has been studied and validated by several research groups [50,51,52,53,54]. Based on the presented technical data and the current clinical developments of autologous and allogeneic cell-based solutions for knee chondral lesion management, a pilot clinical trial is being devised around the therapeutic FE002 progenitor cell source. Therein, two cytotherapeutic formulation options may tangibly be considered, namely the bioengineered graft presented herein (i.e., chondrogenically induced cells on Chondro-Gide scaffolds) and an injectable FE002 cell suspension to be used in a setup similar to second-generation ACI [48,50]. Therein, injectable FE002 primary chondroprogenitor suspensions may be simply obtained for additionally rationalized manufacturing and clinical workflows.

## 5. Conclusions

The aim of the present study was to perform manufacturing process benchmarking and parallel functional qualification for matrix-associated autologous and allogeneic approaches to large knee (osteo)-chondral defect cytotherapeutic management. Our experimental results confirmed that both types of bioengineered constructs could be manufactured using overlapping and optimized GMP-transposable processes. Specifically, the obtained constructs were characterized by comparable quality- and functionality-related attributes (e.g., *COL2* and *ACAN* induction, GAG deposition, Chondro-Gide scaffold maturation), where quantitative results were relatively superior in the allogeneic sample groups. Based on the existing evidence on the use of HAC/Chondro-Gide combinations, this study confirmed that allogeneic FE002 primary chondroprogenitors were compatible with the Chondro-Gide scaffold and that they could form a functionally sound (i.e., 3D chondrogenic gene induction, ECM deposition) finished product. Additionally, complementary in vitro safety data enabled us to further characterize FE002 primary chondroprogenitors from a preclinical safety viewpoint. The presented data were specifically considered for establishing the rationale around an autologous to allogeneic cell-based orthopedic protocol transposition. These undertakings were based on a gap analysis between the autologous and allogeneic protocols, on the reported functional comparability at the finished product level, and on risk analyses for the use of FE002 primary allogeneic biologicals. Considering the specific discussion points about the available body of research on the current cell-based approaches to large knee (osteo)-chondral defect management enabled us to highlight their respective opportunities, advantages, and risks. Overall, building on the available clinical research on ACI, the present study could enable the establishment of an appropriate standard for the further clinical investigation of FE002 allogeneic cell-based orthopedic protocols.

## Figures and Tables

**Table 1:** Parametric benchmarking of the manufacturing processes for the autologous and the allogeneic protocols. Manufacturing process technical benchmarking may highlight the specific points that require additional data and specific risk analyses for the allogeneic protocol based on a gap analysis and using the approved autologous protocol as a baseline. AHS, autologous human serum; DMEM, Dulbecco’s modified Eagle medium; ECM, extracellular matrix; FBS, fetal bovine serum; HAC, human articular chondrocytes; HPL, human platelet lysate; NA, non-applicable.

Parameter/Specification		Autologous Protocol	Allogeneic Protocol	Purpose/Targets	Resource Requirements ^1^ (Allogeneic vs. Autologous)
Regulatory Status of the Protocol for Clinical Investigational Use		Approved ^2^ (Swissmedic)	Pending Submission	NA	Increased
1. Cellular Active Substance	Cellular Active Substance Proliferation Medium	DMEM–Ham’s F12; L-glutamine; HPL 10%	DMEM; L-glutamine; FBS 10%	Obtention of ≥40 × 10^6^ cells for a cellular active substance lot	Decreased
	Cellular Active Substance Lot Cryopreservation	Yes; Liquid nitrogen	Yes; liquid nitrogen	Maintenance of appropriate biological functionalities	Conserved
	Cellular Active Substance Lot Processing for Scaffold Seeding	HACs expanded once in 2D before scaffold seeding	FE002 primary chondroprogenitors directly seeded after initiation	Use of cellular active substance materials with optimal quality and functionality attributes	Decreased
2. Finished Cytotherapeutic Product	Matrix/Scaffold	Chondro-Gide ≤ 20 cm^2^	Chondro-Gide ≤ 20 cm^2^	Use of cyto-/bio-compatible scaffold of appropriate dimensions with appropriate functionalities	Conserved
	Cell Seeding Density on the Scaffold	[2 ± 0.5] × 10^6^ cells/cm^2^	[2 ± 0.5] × 10^6^ cells/cm^2^	Obtention of homogeneous scaffold cell seeding	Conserved
	Chondrogenic Induction Medium Composition	DMEM–Ham’s F12; HPL 10%; L-glutamine; ascorbic acid 0.025 mg/mL; TGF-β1 10 ng/mL; Insulin 10 μg/mL; dexamethasone 100 nM	DMEM; L-glutamine; ITS; TGF-β3 10 ng/mL; VitCp 82 µg/mL; dexamethasone 10 nM	Induction of chondrogenic genes and ECM deposition by viable cells	Conserved
	Chondrogenic Induction Time-Period	16 ± 4 days	14 ± 2 days	Induction of chondrogenic genes and ECM deposition by viable cells	Decreased
	Transport Medium Composition	NaCl 0.9%; AHS 20%	NaCl 0.9%	Maintenance of appropriate physical and biological functionalities	Decreased
	Finished Product Validity Period	6 h after end of manufacture	6 h after end of manufacture	Maintenance of appropriate physical and biological functionalities	Conserved

^1^ Combination of global financial, regulatory, material, and scientific resources. ^2^ NCT05651997 clinical trial, Lausanne and Fribourg, Switzerland.

**Table 2:** Control parameters for the autologous and allogeneic finished products (i.e., in-process indirect assessments and endpoint direct assessments). AB, Alcian Blue; ACAN, aggrecan; C, conforming; COL 2, collagen II; DMMB, dimethylmethylene blue; ECM, extracellular matrix; GAG, glycosaminoglycan; h, hours; HE, hematoxylin and eosin; MTT, 3-(4,5-dimethylthiazol-2-yl)-2,5-diphenyltetrazolium bromide; NC, non-conforming; PBS, phosphate-buffered saline; RT-PCR, real-time polymerase chain reaction.

Control/Assessment Type	Control Parameters	Control Methods	Targets/Acceptance Criteria	Autologous Protocol Assessment		Allogeneic Protocol Assessment	
				C	NC	C	NC
1. Endpoint Direct Assessment ^1^ of Finished Product Lot	Chondrogenic Gene Induction in 3D	RT-PCR	Induction of *COL2* and *ACAN*	✓	-	✓	-
	Cartilage GAG Presence (Total) in 3D	DMMB	Cartilage GAG presence ≥ 0.25 µg/mg	✓	-	✓	-
	Cartilage ECM Presence (Histology) in 3D	HE	Presence of staining	✓	-	✓	-
		ACAN	Presence of staining	✓	-	✓	-
		AB	Presence of staining	✓	-	✓	-
	Cellular Viability in 3D	MTT	Presence of MTT signal	✓	-	✓	-
	Homogeneity of Cell Presence Across the Construct	MTT signal homogeneity; Histology	Homogeneous staining of the whole surface of the construct	✓	-	✓	-
	Cells and Synthetized ECM Localized in 1 Layer of the Construct	MTT; Histology	Presence of cells and ECM in one layer of the construct (absent from the other layer)	✓	-	✓	-
	Cells Localized in Lacunae	Histology	Presence of cells in the lacunae	✓	-	✓	-
	Cellular Morphology	Histology	Rounded cellular morphology	✓	-	✓	-
	Homogeneous ECM Presence Across the Construct	Histology	Homogeneous presence of ECM across the construct	✓	-	✓	-
	Significant ECM Deposition Within the Construct	Histology	Significant ECM deposition in one layer of the construct	✓	-	✓	-
2. In-Process Indirect Assessment ^2^ of Finished Product Lot	Cell Viability in Monolayer Control Cultures	Cell enumeration; Operator assessment	Cellular viability ≥ 75% before control plate seeding; limited amounts of floating dead cells; induction medium consumption	✓	-	✓	-
	Cellular Adhesion in Monolayer Control Cultures	Operator assessment	Presence of ≥60% adherent cells 24 h after seeding; absence of significant cellular detachment	✓	-	✓	-
	Cellular Proliferation in Monolayer Control Cultures	Operator assessment	Appropriate proliferative cellular morphology adoption, proliferation rate, and proliferation homogeneity in monolayer	✓	-	✓	-
	Cellular Population Purity in Monolayer Control Cultures	Operator assessment	Absence of observable cell sub-population presence	✓	-	✓	-

^1^ Destructive control process performed directly on the finished product, requires dedicated replicate production. ^2^ Non-destructive control process performed on cell recovery control cultures or on manufacturing process retention samples.

**Table 3:** Autologous and allogeneic finished product attributes used for the parametric description and multi-phasic control of the in vitro manufacturing process. CQA, critical quality attribute; ECM, extracellular matrix; IPC, in-process control; PPC, post-process control.

Parameter Type	Control Parameters	IPC ^1^	PPC ^2^	Process Development /Validation ^3^	Release Criterion
1. Quality	Cell viability (monolayer recovery)	✓	-	✓	✓
	Cell viability (3D)	-	✓	✓	✓
	Cell proliferation rate (monolayer recovery)	✓	-	✓	✓
	Endpoint cell yield (monolayer recovery)	✓	-	✓	-
	Cell morphology (3D)	-	✓	✓	-
	Localization of cells in the lacunae	-	✓	✓	-
2. Purity	Microscopic cell morphology assessment (monolayer recovery)	✓	-	✓	✓
	Cell proliferation rate (monolayer recovery)	✓	-	✓	✓
3. Efficacy	Cell viability (3D)	-	✓	✓	✓
	Chondrogenic gene induction (3D)	-	✓	✓	-
	Cartilage-specific ECM synthesis and deposition in 3D	-	✓	✓	-
	Macroscopic change in construct color and rigidity	✓	✓	✓	✓
4. Safety	In vitro and in vivo tumorigenicity assessment; telomerase activity quantification; cell type senescence assessment; karyotyping; literature review	-	-	✓	-
	Microbiological safety (manufacturing system)	✓	✓	✓	✓
	Microbiological safety (raw/starting materials, retention samples)	✓	✓	✓	✓
	Microbiological safety (finished product lot)	-	✓	✓	✓
5. Stability	Finished product CQA maintenance after storage	-	✓	✓	-
	Finished product CQA maintenance after transport	-	✓	✓	-

^1^ Performed on cell recovery control cultures or on manufacturing process retention samples. ^2^ Performed on the finished product or on manufacturing process retention samples. ^3^ These activities are characterized by the enhanced scope and granularity of their technical validation compared to the routine finished product release criteria.

**Table 4:** Parametric grading table of allogeneic finished products after 6 h of transport and storage. AB, Alcian Blue; ACAN, aggrecan; DMMB, dimethylmethylene blue; GAG, glycosaminoglycan; HA, hyaluronic acid; HE, hematoxylin and eosin; MTT, 3-(4,5-dimethylthiazol-2-yl)-2,5-diphenyltetrazolium bromide; NaCl, sodium chloride; PBS, phosphate-buffered saline.

Parameter	Controls	Targets/Acceptance Criteria	Endpoint Construct Grading ^1^		
			NaCl	OPTIMEM	HA-PBS
Cellular Viability	MTT	Presence of viable cells on the constructs	+++	+++	+++
Cellular Repartition	MTT/HE	Homogeneous viable cell repartition on one side of the scaffold	+++	+++	+++
Construct Morphology	Operator Assessment	Specific macroscopic change in construct color and rigidity	+++	+++	+++
GAG Content	DMMB	Maintenance of total GAG contents	++	+++	+++
Aggrecan Presence	Histology	Maintenance of positive ACAN staining on one side of the construct	+++	+++	+++
Alcian Blue Staining	Histology	Maintenance of positive AB staining on one side of the construct	+++	+++	+++

^1^ Semi-quantitative grading was performed using the abbreviated nomenclature presented hereafter. (++) = satisfactory; (+++) = optimal.

## Data Availability

The data presented in this study are available from the corresponding authors upon written and reasonable request.

## Supplementary Materials

The following supporting information can be downloaded at https://www.mdpi.com/article/10.3390/pharmaceutics15092333/s1: Figure S1: Comparative cellular proliferation assays; Figure S2: Comparative cellular proliferation curves; Figure S3: Cellular senescence detection assays; Figure S4: Soft agarose colony formation assay; Figure S5: Comparative telomerase activity quantification assay; Figure S6: Cellular active substance stability studies; Figure S7: Finished cytotherapeutic product manufacturing steps; Figure S8: Comparative technical and clinical workflow for the autologous and allogeneic protocols; Figure S9: Finished product suture tests; Table S1: Proteomic characterization results of FE002 allogeneic cellular materials; Table S2: Established cryopreserved cellular active substance quality attributes; Table S3: Established finished product quality attributes; Table S4: Risk analysis matrix for human FE002 primary chondroprogenitor cell type establishment; Table S5: Risk analysis matrix for human FE002 primary chondroprogenitor cell banking; Table S6: Specific risk analysis matrix established for the assessment of the microbiological safety of FE002 primary chondroprogenitors; Table S7: General risk analysis matrix established for the finished products.
