# Supplementary material for: Autologous and Allogeneic Cytotherapies for Large Knee (Osteo)Chondral Defects: Manufacturing Process Benchmarking and Parallel Functional Qualification
Source: Pharmaceutics. 2023 Sep 16;15(9):2333. doi: 10.3390/pharmaceutics15092333 (PMC10536774; doi:10.3390/pharmaceutics15092333)
Supplement: Supplementary file 1 [file pharmaceutics-15-02333-s001.zip › pharmaceutics-2589060-Supplementary.pdf]

pharmaceutics

Supplementary Materials:

Autologous and Allogeneic Cytotherapies for Large Knee (Osteo)Chondral Defects: Manufacturing Process Benchmarking and Parallel Functional Qualification

Virginie Philippe, Annick Jeannerat, Cédric Peneveyre, Sandra Jaccoud, Corinne Scaletta, Nathalie Hirt-Burri, Philippe Abdel-Sayed, Wassim Raffoul, Salim Darwiche, Lee Ann Applegate, Robin Martin and Alexis Laurent

1. List of Abbreviations

AB	Alcian blue
ACAN	aggrecan
ACI	autologous chondrocyte implantation
AD-MSC	adipose-derived mesenchymal stem cells
AHS	autologous human serum
aNC	autologous nose cartilage
ATMP	advanced therapy medicinal product
BCA	bicinchoninic acid
CAM	chorioallantoic membrane model
cATMP	combined advanced therapy medicinal product
CD	cluster of differentiation
CER-VD	Commission cantonale d'éthique de la recherche sur l'être humain-Vaud
cGMP	current good manufacturing practices
CH	Helvetic Confederation
CHUV	centre hospitalier universitaire vaudois
COL	collagen
COMP	cartilage oligomeric matrix protein
CPP	critical process parameter
CQA	critical quality attribute
CT	cycle threshold
DMEM	Dulbecco's modified Eagle medium
DMMB	dimethylmethylene blue assay
DMSO	dimethyl sulfoxide
DNA	deoxyribonucleic acid
DTT	dithiothreitol
EC	European Commission
ECM	extracellular matrix
EDTA	ethylenediaminetetraacetic acid
EMA	European Medicines Agency
EtOH	ethanol
EU	European Union
EU	endotoxin unit
FBS	fetal bovine serum
FDA	US Food and Drug Administration
FGF-2	fibroblast growth factor 2
FU	follow-up
GAG	glycosaminoglycan
GAPDH	glyceraldehyde-3-phosphate dehydrogenase

GFP	green fluorescent protein
GLP	good laboratory practices
GMP	good manufacturing practices
h	hour
HA	hyaluronic acid
HAC	human articular chondrocytes
HE	hematoxylin & eosin
HPL	human platelet lysate
ICRS	International Cartilage Regeneration and Joint Preservation Society
IKDC	International Knee Documentation Committee
IL	interleukin
IMP	investigational medicinal product
IMPD	investigational medicinal product dossier
IPC	in-process control
ITS	insulin, transferrin, selenous acid
KOOS	knee injury and osteoarthritis outcome score
KPP	key process parameter
KQA	key quality attribute
MACI	matrix-induced autologous chondrocyte implantation
MACT	matrix-assisted autologous chondrocyte transplantation
MCB	master cell bank
MDa	megaDalton
MFx	microfracture
min	minute
MMP	matrix metalloproteinase
MoA	mechanism of action
MOCART	magnetic resonance observation of cartilage repair tissue
MRI	magnetic resonance imaging
MTT	3-(4,5-dimethylthiazol-2-yl)-2,5-diphenyltetrazolium bromide
NA	non-applicable
NAT	nucleic acid amplification technique
NC	non-conforming
OA	osteoarthritis
OTR	Orthopedics and Traumatology Service
PAF	paraformaldehyde
PBS	phosphate-buffered saline
PCB	parental cell bank
Ph. Eur.	European pharmacopoeia
PMSF	phenylmethanesulfonyl fluoride
PPC	post-process control
QA	quality assurance
QC	quality control
qPCR	quantitative polymerase chain reaction
RAM	risk analysis matrix
RH	relative humidity
RMAT	regenerative medicine advanced therapy
RNA	ribonucleic acid
rpm	rotations per minute
RT	reverse transcriptase
RT-PCR	real-time polymerase chain reaction
SDS	sodium dodecyl sulfate
SOP	standard operating procedure
sTNFRI	soluble tumor necrosis factor receptor 1

TGF-β	transforming growth factor beta
TIMP	tissue inhibitor of metalloproteinases
TNF	tumor necrosis factor
TrSt	standardized transplant product
UK	United Kingdom
USA	United States of America
UTR	Regenerative Therapy Unit
VAS	visual analog scale
VitCp	Vitamin C 2-phosphate
WCB	working cell bank
WOMAC	western Ontario and McMaster Universities arthritis index

2. Supplementary Figures

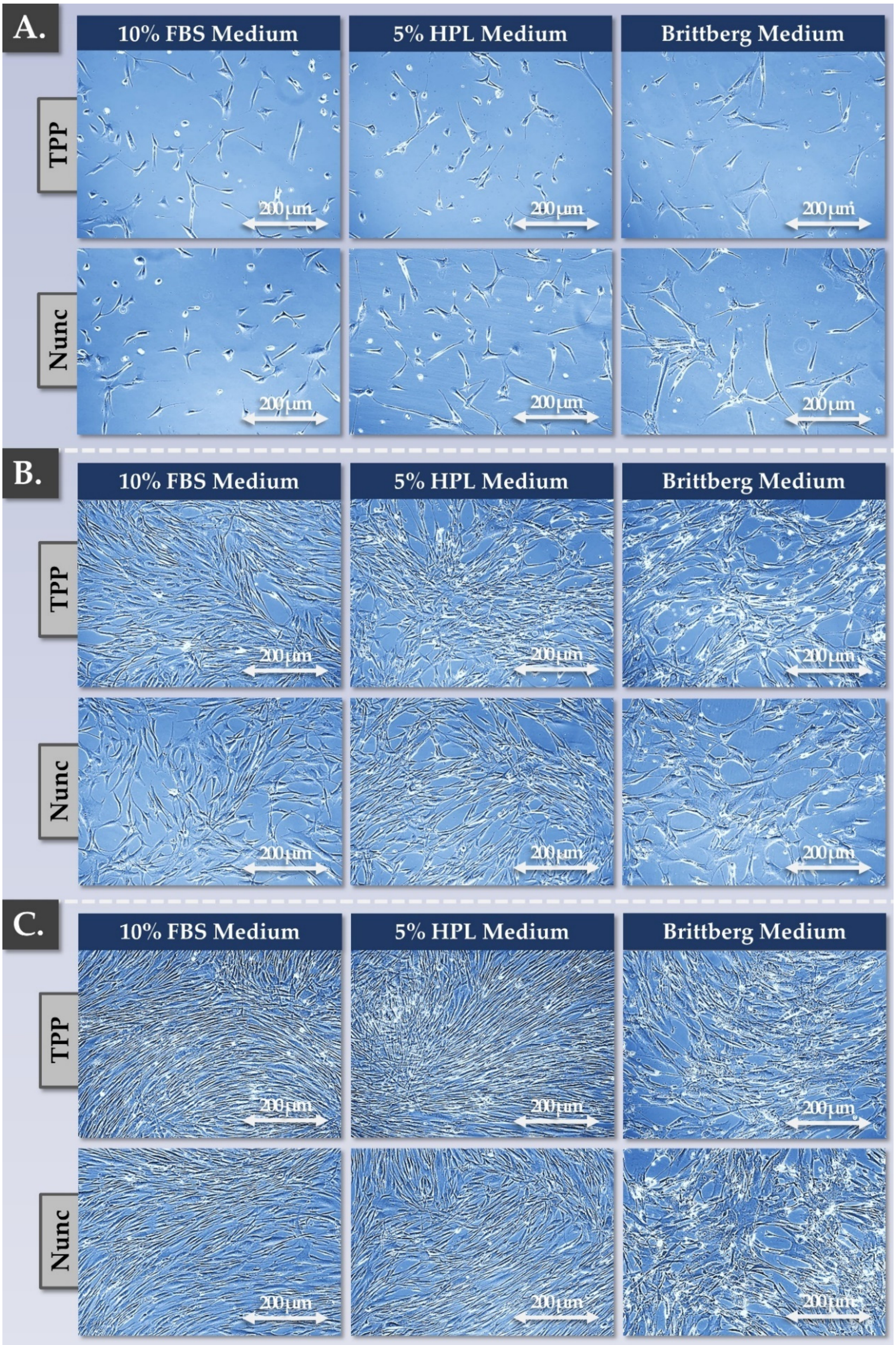

Figure S1. Allogeneic cellular active substance proliferation assay results, using various plastic cell culture surfaces (i.e., TPP or Nunc brands) and cell proliferation medium formulas or supplements (i.e., FBS vs. HPL). (**A**) FE002 primary chondroprogenitors at passage

7, after 3 days in culture. (**B**) Cell monolayer aspect after 7 days in culture. (**C**) Cell monolayer aspect after 10 days in culture. Scale bars = 200 µm. FBS, fetal bovine serum; HPL, human platelet lysate.

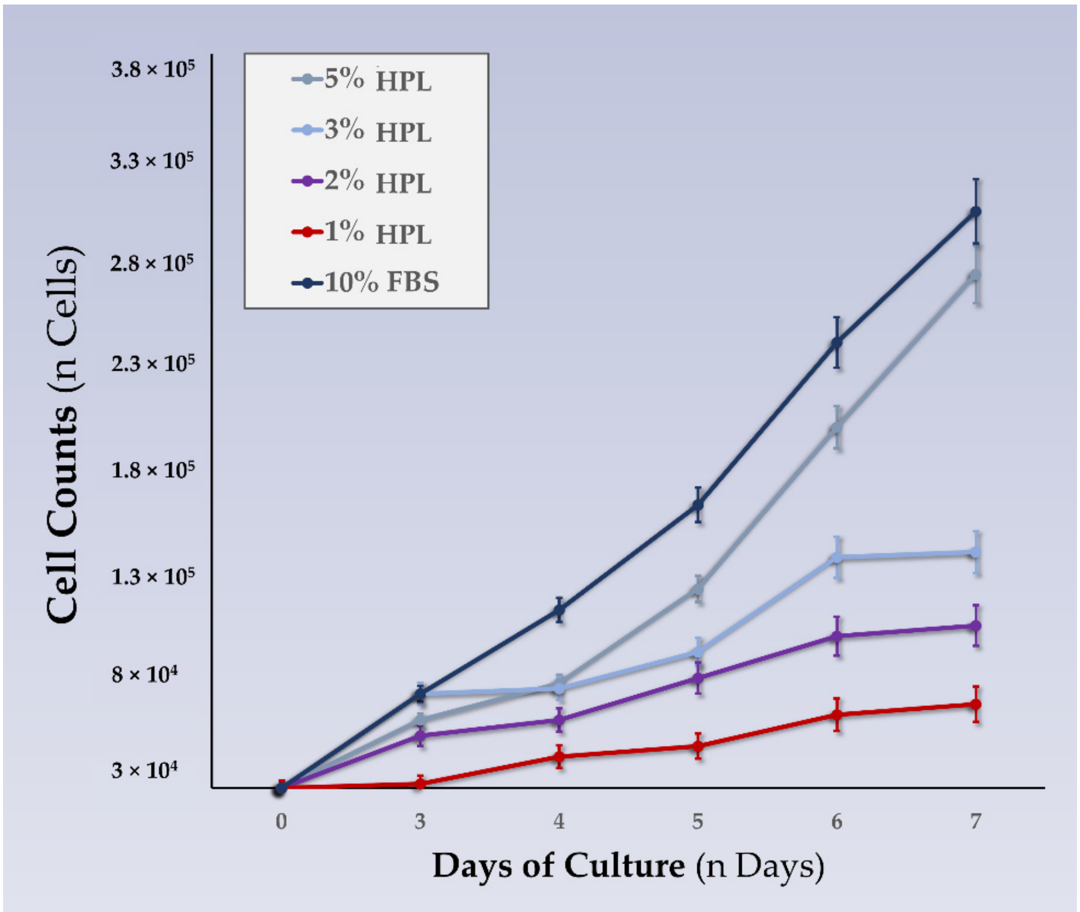

Figure S2. Allogeneic cellular active substance proliferation media supplement qualification results, with comparative proliferation curves of FE002 primary chondroprogenitors. The basal medium was high-glucose DMEM. Results showed that 5% HPL or 10% FBS supplementation resulted in comparable cell proliferation behaviour, as illustrated in Figure S2. DMEM, Dulbecco's modified Eagle medium; FBS, fetal bovine serum; HPL, human platelet lysate.

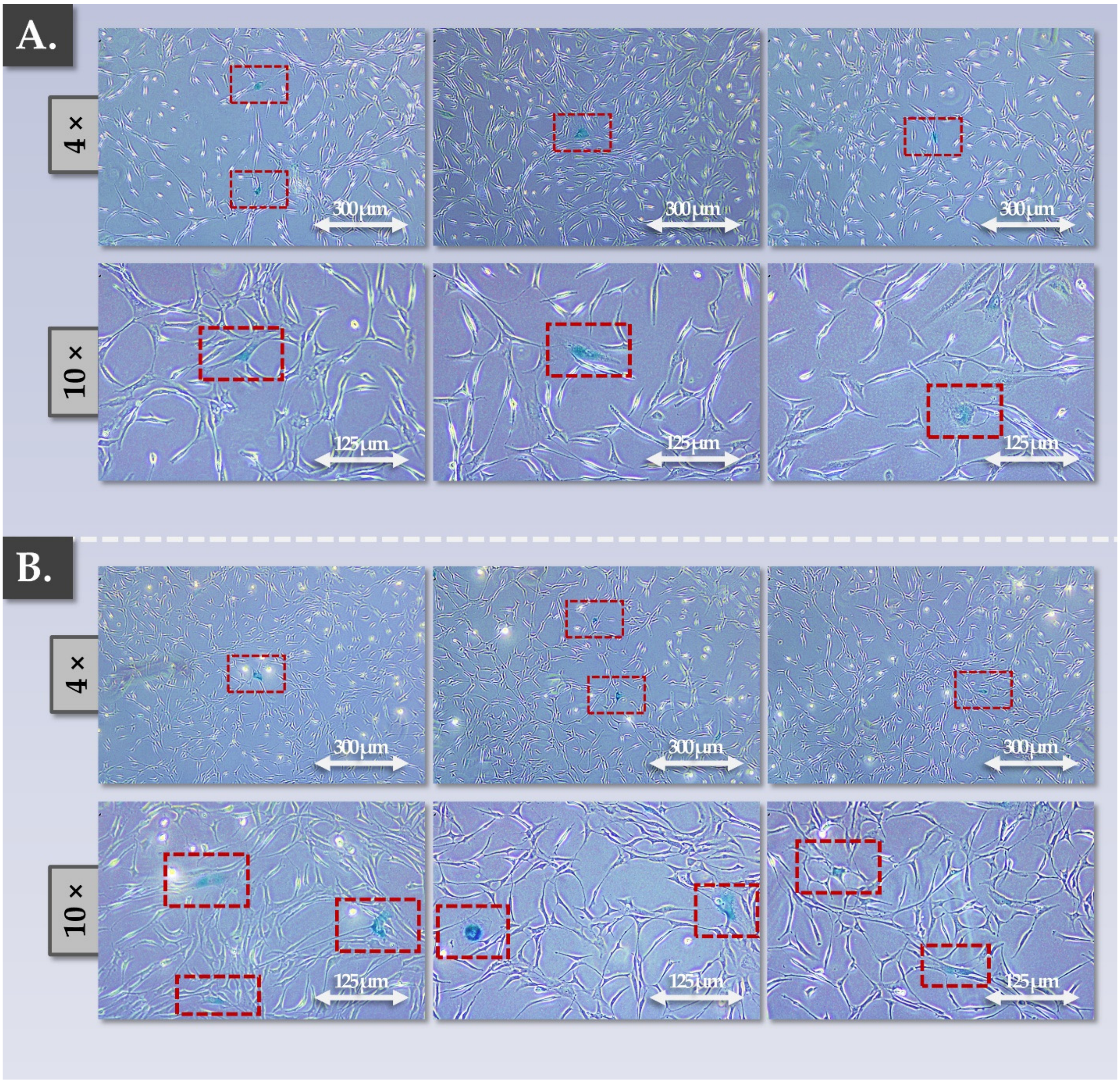

Figure S3. Results of in vitro cellular lifespan characterization assays for the allogeneic cellular active substance. Cellular senescence was detected using β-galactosidase staining of proliferating FE002 primary chondroprogenitors. (**A**) Proliferating FE002 primary chondroprogenitors at passage 7 under various optical enlargements. Cells positive for β-gal staining (i.e., blue stain) were evidenced in red outlines. (**B**) Proliferating FE002 primary chondroprogenitors at passage 8 under various optical enlargements. Cells positive for β-gal staining (i.e., blue stain) were evidenced in red outlines. Scale bars = 125 µm or 300 µm.

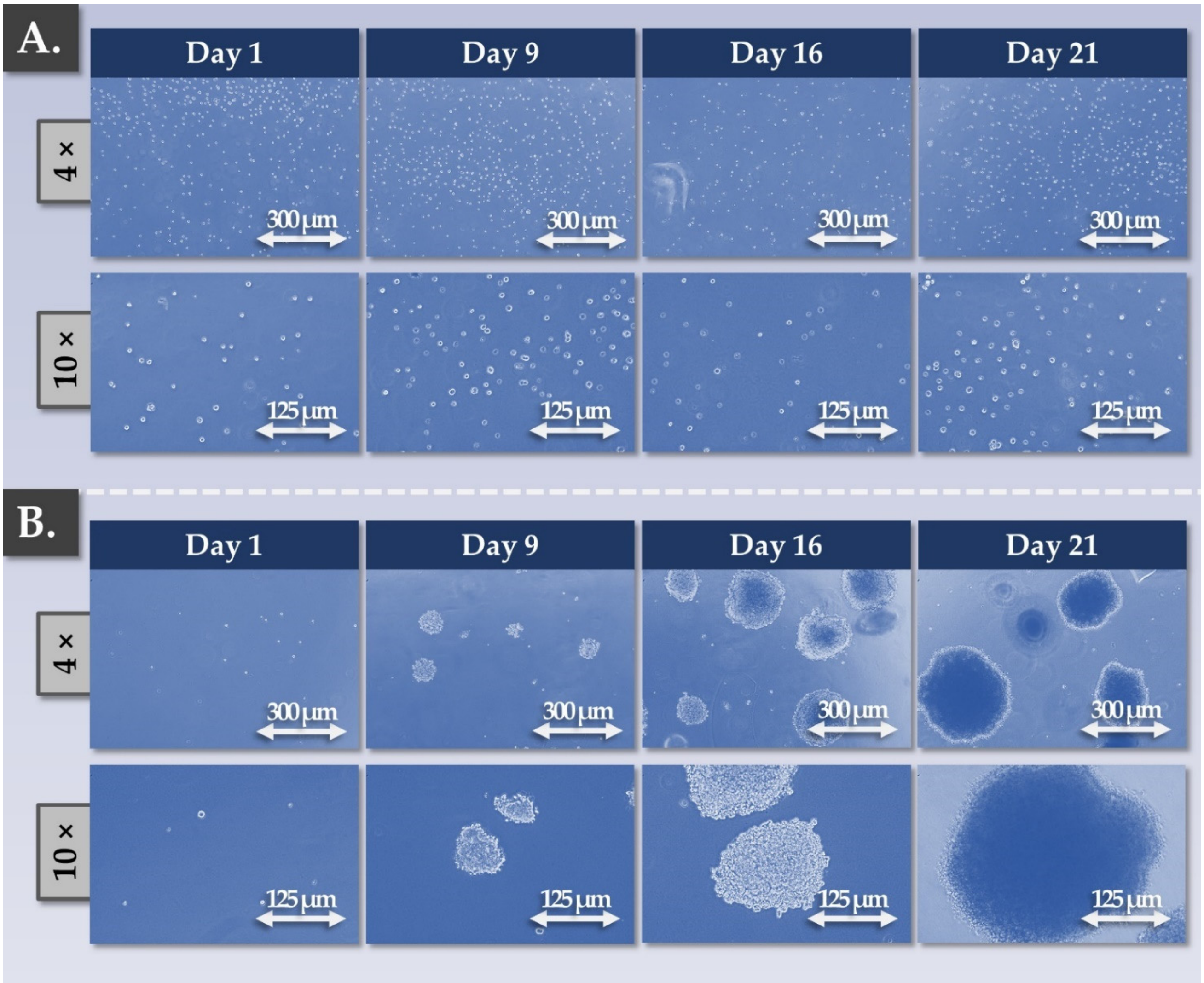

Figure S4. Results of soft agarose colony formation assays for the in vitro characterization of allogeneic cellular active substance safety attributes. (**A**) Multiple timepoints of FE002 primary chondroprogenitor non-adherent in vitro culture in soft agarose under various optical enlargements. No anchorage-independent cellular proliferation was evidenced up to the final timepoint (i.e., day 21). (**B**) Multiple timepoints of HeLa cells (i.e., tumorigenic positive control) non-adherent in vitro culture in soft agarose under various optical enlargements. Important anchorage-independent cellular proliferation was evidenced as early as the second timepoint (i.e., day 9). Scale bars = 125 µm or 300 µm.

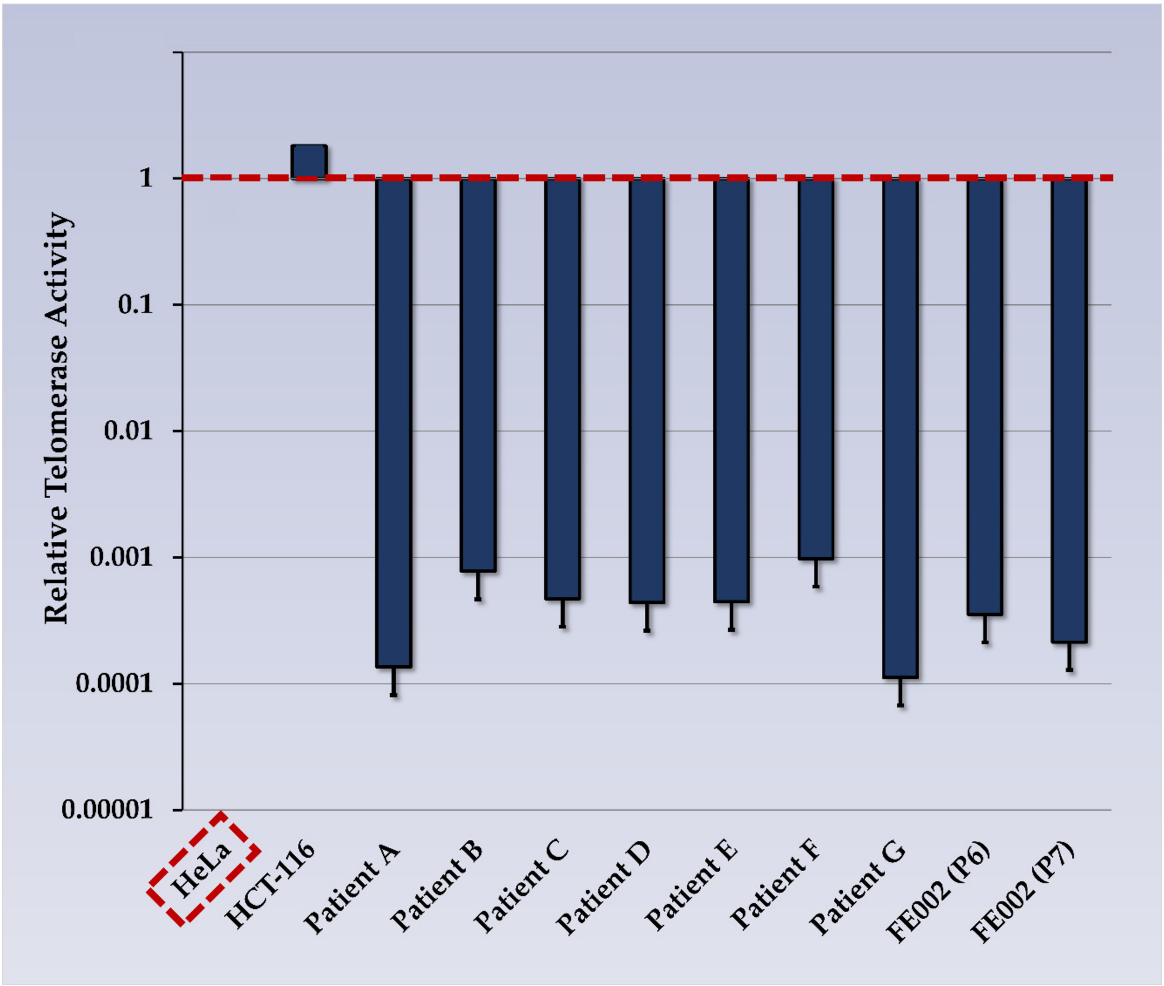

Figure S5. Results of comparative telomerase activity quantification assays for the in vitro characterization of cellular active substance safety attributes. The relative telomerase activity of FE002 primary chondroprogenitors (i.e., passages 6 and 7, n=3 replicates) and of seven primary types of HACs (i.e., n=3 replicates) were expressed as compared to the telomerase activity of HeLa cells. From a quantitative standpoint, mean experimental Ct values were determined at 32.23 (i.e., FE002 primary chondroprogenitors), at 21.88 (i.e., HeLa cells, positive control), and at 23.47 (i.e., telomerase kit internal positive control), respectively. HCT-116 cells were used as an additional positive control and the corresponding telomerase activity was found to be higher than that of the HeLa cells. Overall, no significant difference was found between the mean telomerase activity level of patient primary HAC cell types and that of the allogeneic FE002 primary chondroprogenitors. Ct, cycle threshold; HAC, human articular chondrocytes.

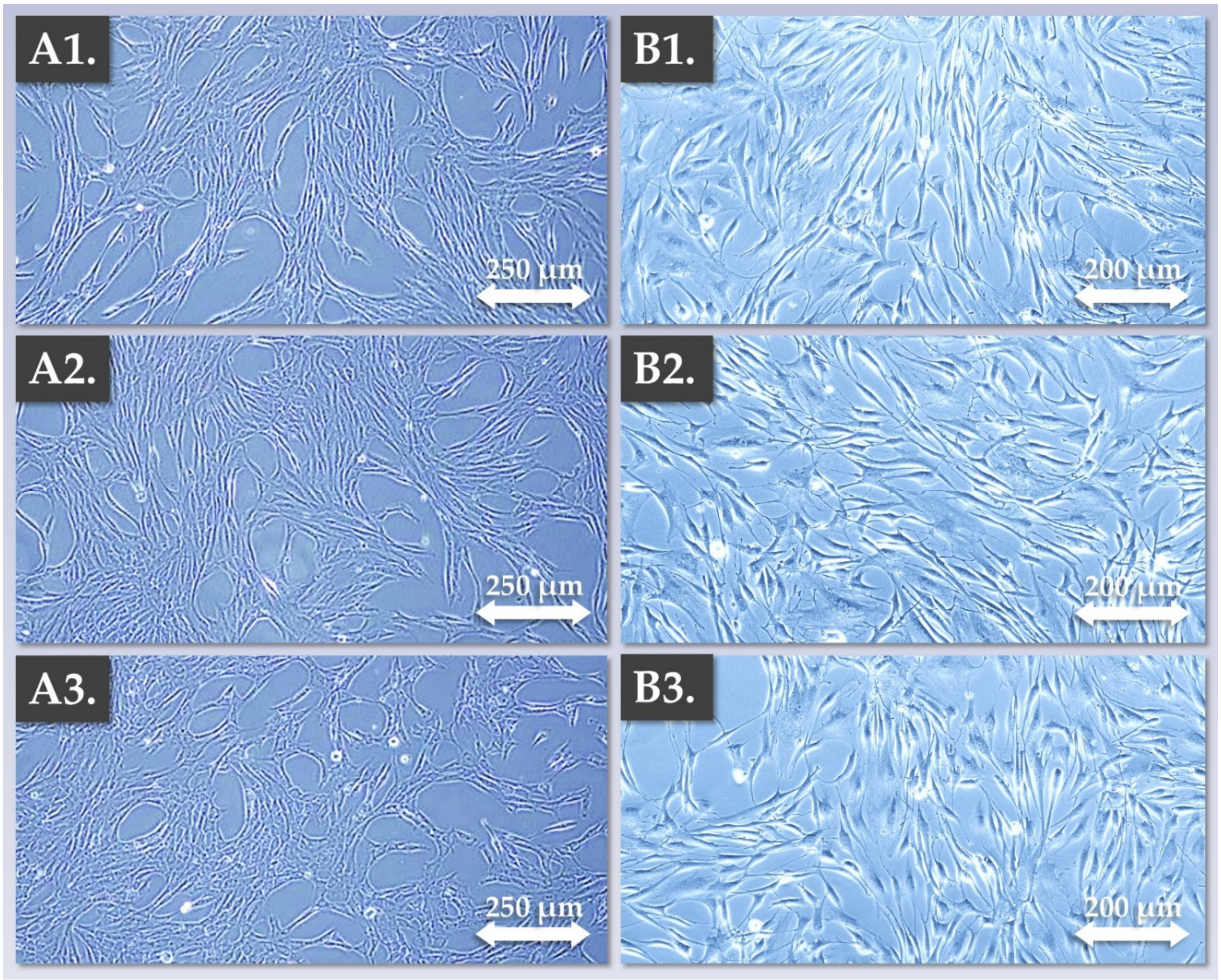

Figure S6. Results of cellular active substance stability studies during cryopreservation in DMSO-based solutions and long-term storage in liquid nitrogen tanks. The cells were initiated and cultured as described for the cellular active substance in vitro manufacturing activities. (**A1**–**A3**) Representative imaging of recovery control assays for HACs, following three years of cryopreservation and cryostorage. (**B1**–**B3**) Representative imaging of recovery control assays for FE002 primary chondroprogenitors, following three years of cryopreservation and cryostorage. The results confirmed that all of the investigated materials conformed to the critical quality attributes (CQA) specified for the cellular active substance, thereby validating the storage conditions and the validity period for a 3-year time-period. Scale bars = 200 µm or 250 µm. CQA, critical quality attribute; DMSO, dimethyl sulfoxide; HAC, human articular chondrocytes.

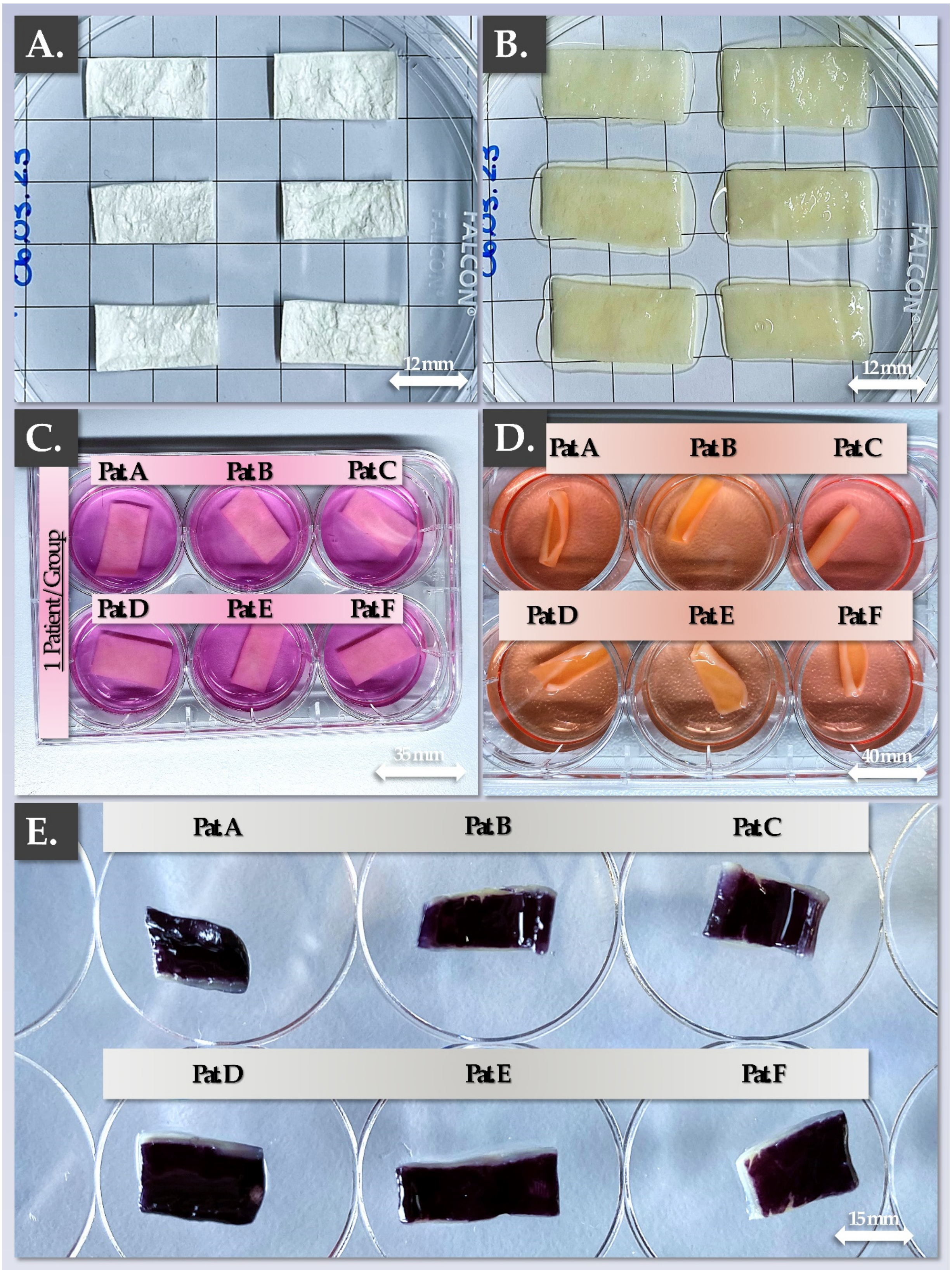

Figure S7a. Illustrative overview of the autologous cytotherapeutic product manufacturing process, represented with the main technical steps performed over a two-week time-period. (**A**) Presentation of the dry Chondro-Gide scaffold following aseptic unpackaging and cutting. (**B**) Presentation of the constructs following wetting with HPL. A slight increase in membrane size is observed upon wetting. (**C**) Presentation of the constructs at the beginning of the in vitro incubation phase. (**D**) Presentation of the constructs before endpoint harvest. Macroscopic changes (i.e., construct color and shape) are observable and the induction medium was consumed by the cells (i.e., observed medium acidification). (**E**) Endpoint MTT control showing conforming constructs (i.e.,

homogeneous construct colonization by viable cells, presence of cells on a single construct side, comparable function between the six patient groups). Scale bars = variable values. HPL, human platelet lysate.

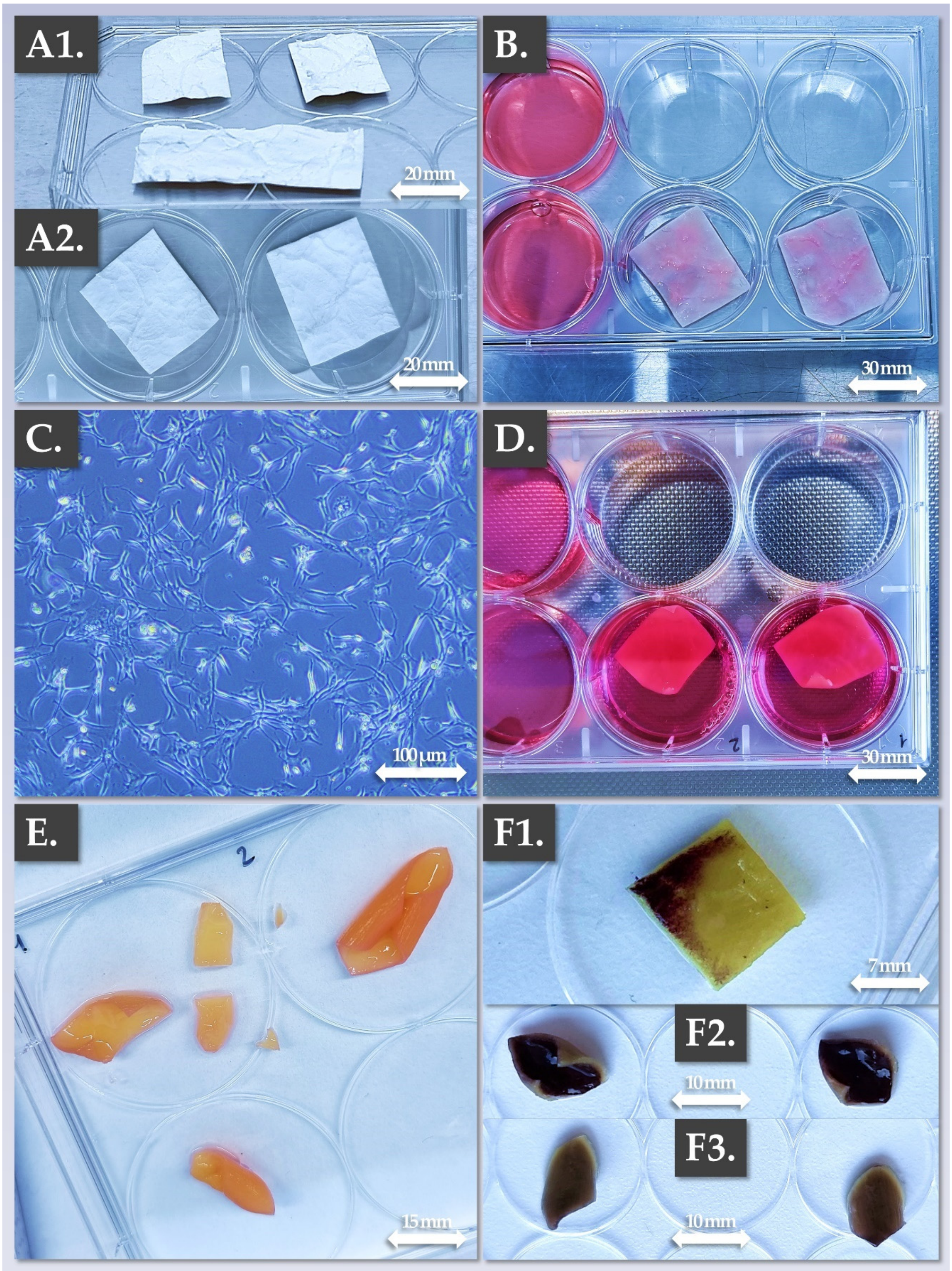

Figure S7b. Illustrative overview of the allogeneic cytotherapeutic product manufacturing process, represented with the main technical steps performed over a two-week time-period. (**A1**,**A2**) Presentation of the dry Chondro-Gide scaffold following aseptic unpackaging and cutting. (**B**) Presentation of the constructs following FE002 cell seeding. A slight increase in membrane size is observed upon wetting. (**C**) Representative microscopic imaging of a cell recovery control well, displaying proliferating FE002

primary chondroprogenitors. (**D**) Presentation of the constructs during the in vitro incubation phase. (**E**) Presentation of the constructs after endpoint harvest and manual cutting. Macroscopic changes (i.e., construct color and shape) are observable. (**F1**) Endpoint MTT control, showing a non-conforming construct (i.e., inhomogeneity of cellular colonization and weak signal). (**F2**,**F3**) Endpoint MTT control showing conforming constructs (i.e., homogeneous construct colonization by viable cells, presence of cells on a single construct side). Scale bars = variable values.

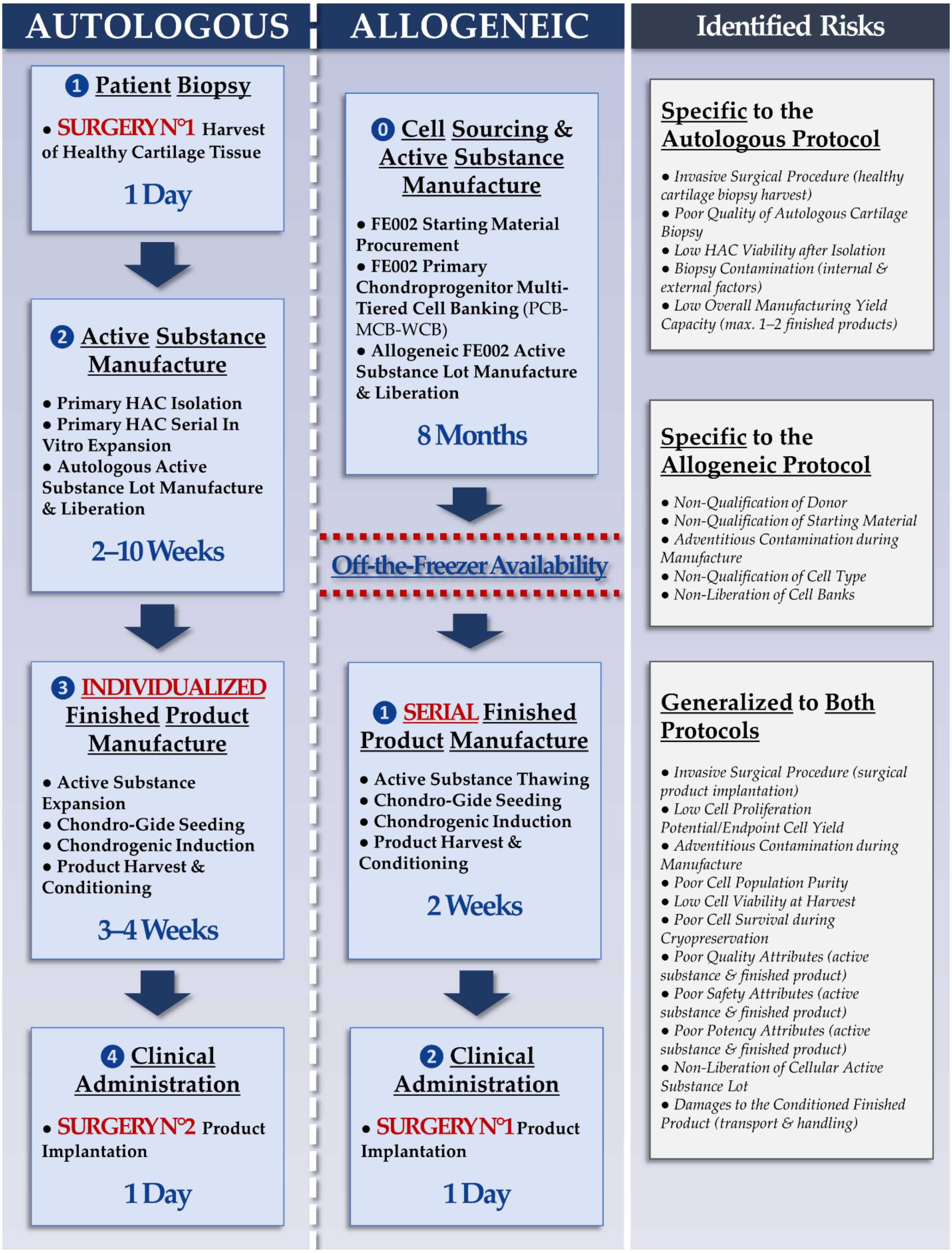

Figure S8. Comparative technical and clinical workflow for the autologous and the allogeneic protocols for the cytotherapeutic treatment of large knee chondral/osteochondral defects in Lausanne, Switzerland. Major identified risks are listed for each protocol and for both protocols, respectively. Importantly, the use of the allogeneic protocol enables to lower the operative burden (i.e., no autologous biopsy harvest procedure, no blood draw). Furthermore, important organisational gains are procured by the use of the allogeneic protocol, as the FE002 cellular active substance expansion phases are temporally distinct from finished product manufacturing (i.e., progenitor cells available off-the-freezer). HAC, human articular chondrocytes; MCB, master cell bank; PCB, parental cell bank; WCB, working cell bank.

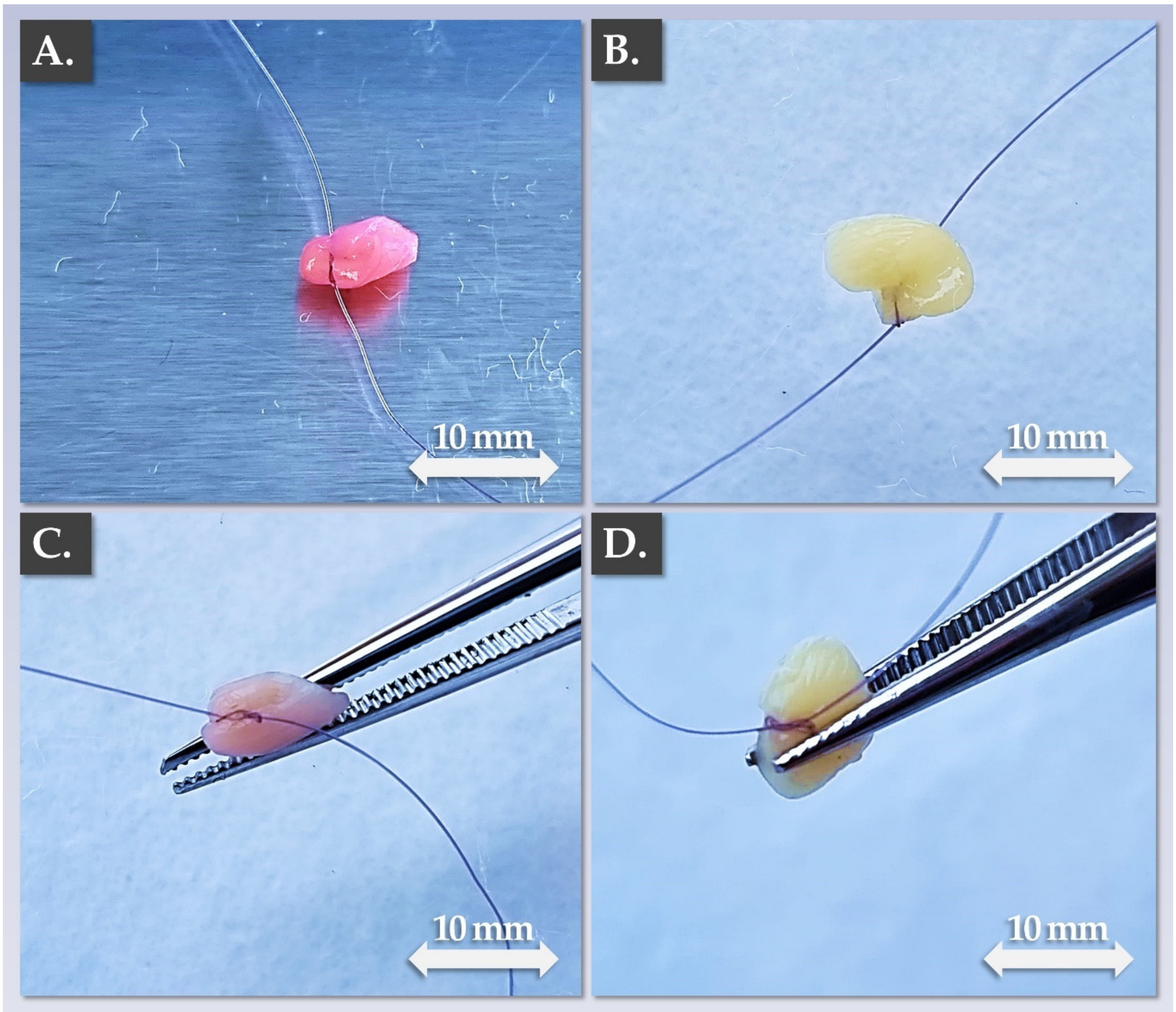

Figure S9. Results of the allogeneic finished product suture tests, confirming construct integrity maintenance during normal processing. (**A**,**C**) Presentation of the sutured constructs directly following harvest from the manufacturing system (i.e., chondrogenic induction vessels). It was confirmed that construct handling, suturing, and gentle mechanical challenging did not compromise the structural integrity of the constructs. (**B**,**D**) Presentation of the sutured constructs following conditioning in transport medium and after submission to the standardized transport protocol (i.e., at the end of the finished product validity period). In the experimental setup, no differences were recorded in finished product attributes before and after the conditioning and transport step, thereby validating the retained specifications. Scale bars = 10 mm.

3. Supplementary Tables

Table S1. Proteomic characterization results of the bulk allogeneic cellular active substance materials (i.e., FE002 primary chondroprogenitor cell lysate, passage 6). The 20 most abundant proteins were reported hereunder, in a decreasing order of appearance, as classified by relative detected quantities. The relative protein quantity, as detected and normalized to the total protein content of the sample soluble fraction, was expressed in pg/mg.

Abbreviated Protein Name	Full Protein Name	Normalized Relative Protein Quantity in the Cell Lysate Soluble Fraction (pg/mg)
TIMP-2	Tissue inhibitor of metalloproteinases 2	15,898.01
MMP-2	Matrix metalloproteinase 2	13,086.77
sEGFR	Soluble epidermal growth factor receptor	3,801.24
TIMP-1	Tissue inhibitor of metalloproteinases 1	1,959.67
sgp130	Soluble gp130	1,504.70
FGF-2	Fibroblast growth factor 2	1,323.40
HGF	Hepatocyte growth factor	433.77
MMP-13	Matrix metallopeptidase 13	410.50
sTNFRI	Soluble tumor necrosis factor receptor type I	409.61
IL-1RA	Interleukin-1 receptor antagonist protein	320.12
Follistatin	Activin-binding protein	299.14
VEGF-A	Vascular endothelial growth factor A	195.38
FGF-1	Fibroblast growth factor 1	170.22
MMP-7	Matrix metallopeptidase 7	117.33
MCP-1	Monocyte chemoattractant protein 1	97.71
MDC	Monocyte-derived chemokine	44.21
VEGF-C	Vascular endothelial growth factor C	44.16
sIL-6R	Soluble IL-6 receptor alpha	43.27
INF-β	Interferon-beta	41.00
sVEGFR3	Soluble vascular endothelial growth factor receptor 3	40.71

Table S2. Established cryopreserved cellular active substance (i.e., FE002 primary chondroprogenitors or patient primary HACs) quality attributes, which were specified as key quality attributes (KQA) or as critical quality attributes (CQA). CQA, critical quality attribute; EU, endotoxin units; HAC, human articular chondrocytes; KQA, key quality attribute.

Cellular Active Substance Quality Attributes	Quality Attribute Type	Requirements for a Cellular Active Substance Lot (Cumulative)
• **Cellular Active Substance Identity**	CQA	Appropriate cellular morphology and behaviour in two-dimensional culture; appropriate increase in chondrogenic gene expression in three-dimensional culture; appropriate specific gene expression markers.
• **Cellular Active Substance Purity**	KQA	Appropriate cellular morphology and behaviour in two-dimensional culture; appropriate specific gene expression markers.
• **Cellular Active Substance Function**	CQA	Appropriate increase in chondrogenic gene expression in three-dimensional culture.
• **Lot Sterility (bacteria & fungi)**	CQA	Absence of detection for specified and non-specified contaminants or values of detection < to specified thresholds.
• **Lot Sterility (mycoplasma)**	CQA	Absence of detection for specified and non-specified contaminants.
• **Lot Acceptable Endotoxin Level**	CQA	Endotoxin level <0.2 EU/mL.
• **Cellular Viability Maintenance**	CQA	Cellular viability maintenance throughout storage, resulting in cellular viability of ≥80% upon initiation from storage.
• **Cellular Proliferation Capacity Maintenance**	CQA	Possibility of in vitro monolayer adherence and expansion for ≥2 passages.
• **Number of Cells per Cellular Active Substance Container**	KQA	Specified number of cells/container ± 20%.
• **Appropriate Cellular Active Substance Lot Identification**	CQA	Correct labelling of cellular active substance packaging materials.
• **Appropriate Cellular Active Substance Lot Storage**	CQA	Cryogenic storage at temperatures constantly <–145 °C.

**• Appropriate Cellular Active Substance Lot Validity**	CQA	Use of the cellular active substance lot within the specified validity period.

Table S3. Established combination product (i.e., autologous or allogeneic) quality attributes, which were specified as key quality attributes (KQA) or as critical quality attributes (CQA). CQA, critical quality attribute; EU, endotoxin units; KQA, key quality attribute.

**Finished Product Quality Attributes**	**Quality Attribute Type**	**Requirements for a Finished Product Lot** (Cumulative)
**• Formulated Cellular Active Substance Identity**	CQA	Appropriate traceability for formulation of the correct cellular active substance in the finished product.
**• Lot Sterility (bacteria & fungi)**	CQA	Absence of detection for specified and non-specified contaminants or values of detection < to specified thresholds.
**• Lot Sterility (mycoplasma)**	CQA	Absence of detection for specified and non-specified contaminants.
**• Lot Acceptable Endotoxin Level**	CQA	Endotoxin level <0.2 EU/mL.
**• Cellular Viability Maintenance**	CQA	Cellular viability maintenance in the finished product during the whole specified product validity period.
**• Total Cell Dose**	KQA	Specified number of cells/product dose ± 20%.
**• Product Quantity**	KQA	Appropriate product quantity at the time of reconciliation with the medical prescription.
**• Product Administration System**	CQA	Appropriate administration system for the planned surgical operation, as prescribed.
**• Product Lot Identification**	CQA	Correct labelling of product packaging materials and product primary containers.

• **Appropriate Product Lot Storage**	CQA	Ambient temperature (15–25 °C) storage of the finished product lot.
• **Appropriate Product Lot Validity**	CQA	Use of the finished product lot within the specified product validity period.

Table S4. Risk analysis matrix (RAM) for clinical grade human FE002 primary chondroprogenitor cell type establishment (i.e., including starting biological material sourcing). GMP, good manufacturing practices; QC, quality control; RAM, risk analysis matrix; SOP, standard operating procedure.

**Parameters**	**Pre-Mitigation Components of the Risk Analysis**			**Risk Severity** (0–3) [1]	**Risk Likelihood** (0–2) [2]	**Risk Level** (0–2) [3]	**Mitigation Measures** (Cumulative)	**Post-Mitigation Risk Level** (0–2) [4]
	**RISKS**	**CAUSES**	**EFFECTS**					
**Donor Qualification**	① Seropositivity for specified pathogens<br>② Seropositivity for unspecified pathogens<br>③ Presence of specified exclusion criteria	① Inadequate anamnesis<br>② Inadequate testing scheme<br>③ Presence of undetectable or latent infection	① Donor qualification failure<br>② Contaminated cellular active substance<br>③ Cellular active substance qualification failure	3	1	2	① Thorough donor anamnesis<br>② Use of specified donor inclusion & exclusion criteria<br>③ Use of highly specific & sensitive donor screening methods<br>④ Screening at T_0 & at $T_{0+90 \text{ days}}$ to exclude donor seroconversion	0
**Donation Qualification**	① Anatomical or physiological abnormality	① Inadequate donor anamnesis<br>② Presence of undetectable pathological abnormality	① Donation qualification failure	2	1	2	① Extensive donor screening & anamnesis<br>② Pathological examination performed by trained pathologist<br>③ Specific histopathology analyses	0

**Cartilage Biopsy Processing Failure**	❶ Low viability of isolated parent cells ❷ Insufficient parent cell yield	❶ Inadequate biopsy processing	❶ Loss of usable starting materials	**3**	**1**	**2**	❶ Validated biopsy processing SOP ❷ Trained manufacturing personnel	**0**
**Chondrogenic Cell Type Instability**	❶ Non-qualification for in vitro cell manufacturing ❷ Apparition of cell tumorigenicity/toxicity	❶ Spontaneous mutation ❷ Non-adaptation to in vitro cell culture	❶ Critical sustainability problematic ❷ Critical safety problematic	**3**	**1**	**2**	❶ Pilot qualification of cell type for in vitro serial expansion ❷ Evolutive karyotyping scheme ❸ In vitro cell behaviour surveillance; morphology, proliferation rate	**0**
**Low Potential for Cell Banking**	❶ Low cell resistance to cryopreservation ❷ Low cell proliferation potential	❶ High cell sensitivity to cryogenic shock ❷ Inadequate cell culture conditions	❶ Critical quality problematic ❷ Critical sustainability problematic	**2**	**1**	**2**	❶ Monitoring of cell culture quality parameters ❷ Qualification & validation of materials & consumables	**0**

**Contamination of Biological Materials & Cell Banks**	❶ Introduction of extraneous contaminants by reagents, equipment, material, personnel<br>❷ Emergence of latent or transient virus<br>❸ Cross-contamination by a similar cell type<br>❹ Cell population switch	❶ Adventitious agent introduction during manufacture, transport, or storage<br>❷ Inadequate segregation of cell cultures<br>❸ Poor initial cell population purity<br>❹ Inadequate cell manufacturing process<br>❺ Insufficient characterization of cell type	❶ Inadequate cell type in manufactured batch<br>❷ Contamination of manufactured cell batch<br>❸ Critical quality problematic<br>❹ Critical safety problematic	**3**	**1**	**2**	❶ Aseptic biological material procurement environment<br>❷ Class A cell manufacturing environment<br>❸ Selection of qualified & tested materials & reagents<br>❹ Trained manufacturing personnel<br>❺ Environmental controls during open-container manipulations<br>❻ Minimization of open-container & contact processes<br>❼ Use of sterile single-use consumables<br>❽ Retention sample testing<br>❾ Post-production testing & batch qualification/release testing<br>❿ Identity & purity QCs of cultured cell populations	**0**

[1] The risk severity is classified as “0 = acceptable”, as “1 = tolerable”, as “2 = undesirable”, or as “3 = intolerable”. [2] The risk likelihood is classified as “0 = improbable”, as “1 = possible”, or as “2 = probable”. [3] The pre-mitigation risk level is classified as “0 = low”, as “1 = medium”, or as “2 = high”. [4] The post-mitigation risk level is classified as “0 = low”, as “1 = medium”, or as “2 = high”.

Table S5. Risk analysis matrix (RAM) for clinical grade human FE002 primary chondroprogenitor cell banking (i.e., including cellular active substance lot manufacture). GMP, good manufacturing practices; MCB, master cell bank; PCB, parental cell bank; QC, quality control; RAM, risk analysis matrix; SOP, standard operating procedure; WCB, working cell bank.

**Parameters**	**Pre-Mitigation Components of the Risk Analysis**			**Risk Severity** (0–3) [1]	**Risk Likelihood** (0–2) [2]	**Risk Level** (0–2) [3]	**Mitigation Measures** (Cumulative)	**Post-Mitigation Risk Level** (0–2) [4]
	**RISKS**	**CAUSES**	**EFFECTS**					
**Cell Viability**	❶ Loss of cell viability	❶ Inadequate storage or handling<br>❷ Inadequate cryopreservation solution	❶ Reduction of cell manufacturing yield<br>❷ Reduced cell batch quality	**2**	**1**	**2**	❶ Storage temperature stability validation & monitoring<br>❷ Iterative total & viable cell enumeration<br>❸ Monitoring of cell culture quality [5]<br>❹ Rinsing of detached cells in culture<br>❺ Trained manufacturing personnel<br>❻ Validated cell thawing SOP<br>❼ Qualification of cryopreservation solution	**0**

**Cell Bank Storage System Failure**	❶ Critical rise in vial temperature/vial thawing ❷ Catastrophic defect in vial structure or in Dewar storage tank system [6] ❸ Catastrophic defect in cell storage facility	❶ Material & equipment failures ❷ System failures ❸ Absence of storage system or cooling liquid replenishing system redundancies	❶ Loss of vial batch or loss of whole cell banks	**3**	**0**	**1**	❶ Use of qualified primary containers (e.g., polymeric vials) & storage tanks (e.g., on-line or off-line tanks) ❷ Segregation of high-value vials in redundant storage systems/storage facilities ❸ Segregation of high-value vials in redundant storage tanks ❹ Nitrogen level/temperature monitoring & alarms ❺ Critical failure alarms ❻ Regular inspection of storage tanks ❼ Inspection of individual vials at the time of cell initiation	**0**
**Low Potential for Cell Banking**	❶ Low cell resistance to cryopreservation ❷ Low cell proliferation potential	❶ High cell sensitivity to cryogenic shock ❷ Inadequate cell culture conditions (e.g., serum lot)	❶ Critical quality problematic ❷ Critical sustainability problematic	**2**	**1**	**2**	❶ Monitoring of cell culture quality parameters ❷ Qualification of materials & consumables	**0**
**Cross-Contamination or Cell Population Switch**	❶ Cross-contamination by a similar cell type ❷ Cell population switch	❶ Inadequate segregation of cell cultures ❷ Poor initial cell population purity	❶ Inadequate cell type introduced during manufacturing	**3**	**1**	**2**	❶ Iterative identity & purity QCs ❷ Segregation of cell types to specific manufacturing areas & equipment ❸ Use of sterile single-use consumables	**0**

**Functional Loss by the Cellular Active Substance**	❶ Loss of chondrogenic potential ❷ Ineffective product manufacture	❶ Inadequate cell manufacture or storage ❷ Poor cell type functional quality ❸ Expansion at excessive cellular passage levels	❶ Rejection of finished products ❷ Ineffective therapeutic intervention	**3**	**1**	**1**	❶ Monitoring of cell culture quality ❷ Use of qualified & consistent in vitro cell passage levels ❸ Standard functional QCs	**0**
**Adventitious Contamination of PCBs**	❶ Contaminated PCB ❷ Non-qualification & rejection of PCB	❶ Adventitious agent introduction during manufacture, transport, or storage	❶ Loss of the parental cells/PCB ❷ Need for cell type re-establishment from new donation	**3**	**1**	**2**	❶ Trained manufacturing personnel ❷ Class A manufacturing environment ❸ Environmental controls during open-container manipulations ❹ Selection of qualified & tested materials & reagents ❺ Minimization of open-container & contact processes ❻ Use of sterile single-use consumables ❼ Retention sample testing ❽ Post-production PCB qualification	**0**

**Adventitious Contamination of MCBs**	❶ Contaminated MCB<br>❷ Non-qualification & rejection of MCB	❶ Adventitious agent introduction during manufacture, transport, or storage	❶ Loss of large quantities of cells<br>❷ Need for MCB re-establishment from PCB	**3**	**1**	**2**	❶ Trained manufacturing personnel<br>❷ Qualification of source PCB<br>❸ Class A manufacturing environment<br>❹ Environmental controls during open-container manipulations<br>❺ Selection of qualified & tested materials & reagents<br>❻ Minimization of open-container & contact processes<br>❼ Use of sterile single-use consumables<br>❽ Retention sample testing<br>❾ Post-production MCB qualification	**0**
**Adventitious Contamination of WCBs**	❶ Contaminated WCB<br>❷ Non-qualification & rejection of WCB	❶ Adventitious agent introduction during manufacture, transport, or storage	❶ Loss of cell batch<br>❷ Need for WCB re-establishment from MCB	**2**	**1**	**2**	❶ Trained manufacturing personnel<br>❷ Qualification of source MCBs<br>❸ Class A manufacturing environment<br>❹ Environmental controls during open-container manipulations<br>❺ Selection of qualified & tested materials & reagents<br>❻ Minimization of open-container & contact processes<br>❼ Use of sterile single-use consumables<br>❽ Retention sample testing<br>❾ Post-production WCB qualification	**0**

**Cell Type Instability**	❶ Non-qualification for in vitro culture ❷ Apparition of toxicity ❸ Karyotype instability	❶ Karyotype alteration ❷ Cells beyond acceptable in vitro age ❸ Non-adaptation to in vitro culture	❶ Critical sustainability problematic ❷ Critical safety problematic ❸ Disqualification of cell bank	**3**	**1**	**2**	❶ MCB, WCB karyotyping ❷ Qualification of cells for in vitro serial expansion ❸ In vitro toxicity studies	**0**
**Cellular Active Substance Tumorigenicity**	❶ Tumoral proliferation of the cellular active substance	❶ Spontaneous mutation ❷ Cells beyond acceptable in vitro age	❶ Tumor formation	**3**	**0**	**1**	❶ In vitro & in vivo evaluation of cellular active substance tumorigenicity potential	**0**

[1] The risk severity is classified as "0 = acceptable", as "1 = tolerable", as "2 = undesirable", or as "3 = intolerable". [2] The risk likelihood is classified as "0 = improbable", as "1 = possible", or as "2 = probable". [3] The pre-mitigation risk level is classified as "0 = low", as "1 = medium", or as "2 = high". [4] The post-mitigation risk level is classified as "0 = low", as "1 = medium", or as "2 = high". [5] Monitoring includes proliferative cellular morphology, cell adhesion, growth rate, metabolic activity, confluency level, cell monolayer homogeneity, sub-population exclusion, and gross microbiological contamination exclusion. [6] Includes rupture or explosion of vials and catastrophic defects in the liquid nitrogen filling system.

Table S6. Specific risk analysis matrix (RAM) established for the assessment of the microbiological safety (i.e., excluding viruses) of FE002 primary chondroprogenitors (i.e., clinical grade cellular active substance). The microbiological safety (i.e., absence of bacteria, fungi, mycoplasma, endotoxins) of the materials serving for the GMP manufacture of cytotherapeutic products is assessed at the time of the selection of starting, raw, and ancillary materials and testing thereof, during production, and during post-production testing. FBS, fetal bovine serum; GMP, good manufacturing practices; MCB, master cell bank; QC, quality control; RAM, risk analysis matrix; WCB, working cell bank.

**Parameters**	**Pre-Mitigation Components of the Risk Analysis**			**Risk Severity** (0–3) [1]	**Risk Likelihood** (0–2) [2]	**Risk Level** (0–2) [3]	**Mitigation Measures** (Cumulative)	**Post-Mitigation Risk Level** (0–2) [4]
	**RISKS**	**CAUSES**	**EFFECTS**					
**Species of Origin**	❶ Risk of infection by zoonotic pathogens	❶ Inclusion of infected donor materials	❶ Zoonotic contamination of the cellular active substance & infection of patient	**3**	**0**	**1**	❶ Selection of human starting materials<br>❷ Thorough testing for pathogens with human tropism<br>❸ Selection of qualified FBS/trypsin lots	**0**
**Tissue of Origin**	❶ Use of contaminated starting materials	❶ Use of tissue type prone to contamination	❶ Contamination of the cellular active substance<br>❷ Infectious risk for the patient	**3**	**1**	**1**	❶ Selection of tissue with low probability of high contaminant yield<br>❷ Thorough qualification of donor<br>❸ Thorough qualification of biopsy	**0**

**Contamination During Cellular Active Substance Manufacturing**	❶ Introduction of extraneous contaminant by reagents, equipment, material, personnel ❷ Emergence of latent or transient contaminant in culture	❶ Inadequate manufacturing process ❷ Inadequate control process ❸ Insufficient initial characterization of cell type ❹ Absence of purification regimen & terminal sterilization	❶ Contamination of the active substance ❷ Infectious risk for the patient	**3**	**1**	**2**	❶ Trained manufacturing personnel ❷ Qualification of source cell banks ❸ Class A manufacturing environment ❹ Selection of qualified & tested materials & reagents ❺ Environmental controls during open-container manipulations ❻ Minimization of open-container & contact processes ❼ Use of sterile single-use consumables ❽ Retention sample testing ❾ Post-production cell bank testing & qualification ❿ Post-production bulk product & final product testing & qualification	**0**

**Infectivity or Iatrogenesis Potential of Contaminated Cellular Active Substance**	❶ Iatrogenic infection of the patient ❷ Inadequate management of patient pathology	❶ Non-functional or potentially iatrogenic cellular active substance	❶ Patient contamination ❷ No amelioration or worsening or patient health status	**3**	**1**	**2**	❶ Qualification of MCBs/WCBs ❷ Class A manufacturing environment for the cellular active substance ❸ Environmental controls during open-container manipulations ❹ Retention sample testing ❺ Post-production cellular active substance testing & qualification ❻ Post-production bulk product & final product testing & qualification	**0**
**Amount of Cellular Active Substance per Product Dose**	❶ Contamination of the patient with large dose of pathogen	❶ Large dose of cellular active substance per product dose	❶ Higher probability of patient infection & severe consequences	**3**	**1**	**1**	❶ Use of relatively small cellular active substance quantity per product dose ❷ Use of sensitive detection methods for specified contaminants during testing ❸ Use of restrictive pathogen limits and thresholds	**0**
**Process Controls (donor, starting materials, products)**	❶ Failure in implemented process controls ❷ Inadequacy of implemented process controls	❶ Systemic error in implemented controls ❷ Occasional error in implemented controls ❸ Apparition of new unspecified contaminants	❶ Liberation of contaminated cellular active substance batch ❷ Infectious risk for the patient	**3**	**1**	**2**	❶ Iterative update of process controls ❷ Iterative validation of process controls ❸ Implementation of redundant process controls ❹ Process controls implemented at the appropriate stages of GMP manufacture	**0**

**Biosafety Testing Scheme**	❶ Emergence of pathogen undetected in preliminary subcultures	❶ Presence of pathogen in undetectable quantities in parental cell stock	❶ Contamination of the cellular active substance batch<br>❷ Infectious risk for the patient	**3**	**1**	**1**	❶ Iterative update of biosafety testing schemes<br>❷ Implementation of iterative & redundant testing steps<br>❸ Full microbiological quality testing of MCBs/WCBs	**0**

[1] The risk severity is classified as “0 = acceptable”, as “1 = tolerable”, as “2 = undesirable”, or as “3 = intolerable”. [2] The risk likelihood is classified as “0 = improbable”, as “1 = possible”, or as “2 = probable”. [3] The pre-mitigation risk level is classified as “0 = low”, as “1 = medium”, or as “2 = high”. [4] The post-mitigation risk level is classified as “0 = low”, as “1 = medium”, or as “2 = high”.

Table S7. General risk analysis matrix (RAM) established for the allogeneic finished product (i.e., FE002 chondroprogenitors on Chondro-Gide scaffolds), adapted from the EMA Guideline EMEA/CHMP/410869/2006 “Guideline on human cell-based medicinal products”. The specified parameters were established and based on cellular active substance specifications, on finished product specifications, and on the need for maintenance of appropriate critical quality attributes (CQA). CQA, critical quality attributes; EMA, European Medicines Agency; QC, quality control; RAM, risk analysis matrix; WCB, working cell bank.

**Parameters**	**Pre-Mitigation Components of the Risk Analysis**			**Risk Severity** (0–3) [1]	**Risk Likelihood** (0–2) [2]	**Risk Level** (0–2) [3]	**Mitigation Measures** (Cumulative)	**Post-Mitigation Risk Level** (0–2) [4]
	**RISKS**	**CAUSES**	**EFFECTS**					

**Cellular Active Substance Immunogenicity**	❶ Immune reaction of the recipient to the cellular active substance	❶ Immune recognition of the cellular active substance by the recipients' organism	❶ Treatment failure ❷ Iatrogenesis	**3**	**1**	**1**	❶ Exclusion of recipients with specific immunological/allergic risk factors	**0**
**Poor Finished Product Quality**	❶ Insufficient efficacy of the finished product	❶ Low cell viability at scaffold seeding ❷ Cell death during the differentiation process ❸ Poor extracellular matrix induction during the differentiation process ❹ Inadequacy of storage and handling process ❺ Inadequacy of product administration process	❶ Treatment failure	**2**	**1**	**1**	❶ Validation of cell viability & recovery at scaffold seeding ❷ Appropriate QC parameters for finished product release ❸ Short finished product validity period ❹ Appropriate & specified finished product transport & administration modalities ❺ Trained manufacturing personnel & surgeons ❻ Use of a commercial cell scaffold, with documented safety and clinical track-record	**0**

**Level of Cellular Active Substance Manipulation**	❶ Mutagenicity, oncogenicity, or tumorigenicity of the cellular active substance	❶ High manipulation of the cells<br>❷ Extensive in vitro cell culture	❶ Formation of tumors in patients	**3**	**0**	**1**	❶ No genetic manipulation of the cells<br>❷ No immortalization of the cells<br>❸ No use of viral tools for cell manufacture<br>❹ Cell type tumorigenicity assessment in vitro and in vivo<br>❺ Combination product tumorigenicity in vivo assessment	**0**
**Adventitious Contamination during Finished Product Manufacturing**	❶ Introduction of extraneous contaminants by reagents, equipment, material, personnel	❶ Inadequate manufacturing process (i.e., including storage & transport)<br>❷ Inadequate reagents, materials<br>❸ Inadequate control processes<br>❹ Presence of latent virus in materials<br>❺ Absence of purification regimen & finished product terminal sterilization	❶ Contamination of finished product<br>❷ Infectious risk for the patient	**3**	**1**	**2**	❶ Testing and qualification of MCBs/WCBs<br>❷ Trained manufacturing personnel<br>❸ Class A manufacturing environment<br>❹ Selection of qualified & tested materials & reagents<br>❺ Environmental controls during open-container manipulations<br>❻ Minimization of open-container & contact processes<br>❼ Use of sterile single-use consumables<br>❽ Retention sample testing<br>❾ Post-production finished product testing and liberation	**0**
**Surgery-Related Risks**	❶ Surgery-related complications	❶ Negative patient reaction during surgery<br>❷ Introduction of contaminants during the operation<br>❸ Finished product delamination	❶ Infectious risk for the patient<br>❷ Surgery cancellation<br>❸ Treatment failure<br>❹ Patient re-operation	**1**	**1**	**1**	❶ Pre-operation patient qualification<br>❷ Robust qualification & experience of the orthopedic surgeon<br>❸ Patient follow-up post-surgery	**0**

**Finished Product**	❶ Incompatibility between the cellular active substance & the scaffold<br>❷ Formation of toxic degradation products	❶ Biological or chemical incompatibility or reaction between the cellular active substance & the scaffold	❶ Treatment failure<br>❷ Iatrogenesis	**3**	**0**	**1**	❶ Qualification of the scaffold<br>❷ Qualification of the finished product<br>❸ Historical clinical safety data present with similar finished products (autologous chondrocytes)	**0**
**Duration of Exposure to Finished Product**	❶ Chronic toxicity of the product	❶ Extensive exposure to the finished product after implantation in the joint	❶ Treatment failure<br>❷ Iatrogenesis	**3**	**0**	**1**	❶ Physiological product bio-integration<br>❷ Limited number of finished product applications<br>❸ Limited cellular engraftment in patient tissues	**0**
**Availability of Clinical Safety Data and Experience**	❶ Insufficient safety data/experience gathered	❶ First-in-human clinical use of FE002 primary chondroprogenitors	❶ Absence of tangible evidence for retrospective safety evaluation of product	**2**	**0**	**0**	❶ Peer-reviewed scientific publications on in vivo animal models using FE002 primary chondroprogenitors, reporting safe use<br>❷ Peer-reviewed scientific publications on in vitro and in vivo safety of the FE002 cellular active substance & product prototypes<br>❸ Years of multi-centric clinical experience with alternative progenitor cell types (e.g., dermal progenitor fibroblasts) from the same FE002 organ donation, demonstrating safety and efficacy in cutaneous regenerative medicine	**0**

[1] The risk severity is classified as "0 = acceptable", as "1 = tolerable", as "2 = undesirable", or as "3 = intolerable". [2] The risk likelihood is classified as "0 = improbable", as "1 = possible", or as "2 = probable". [3] The pre-mitigation risk level is classified as "0 = low", as "1 = medium", or as "2 = high". [4] The post-mitigation risk level is classified as "0 = low", as "1 = medium", or as "2 = high".
